# Ubiquitination-driven proteostasis remodeling underlies oxidative stress and actin degradation in citrus huanglongbing

**DOI:** 10.1093/plphys/kiag612

**Published:** 2026-08-25

**Authors:** Min Li, Hongkun Qin, Yundan Hu, Bingqian Qin, Zirui Fan, Mingzhuo Li, Yaping Mao, Fajin Liao, Zhigang Ouyang, Huan Chen, Shuo Duan

**Affiliations:** National Navel Orange Engineering Research Center, College of Life Sciences, Gannan Normal University, Ganzhou 341000, China; Jiangxi Provincial Key Laboratory of Pest and Disease Control of Featured Horticultural Plants, Gannan Normal University, Ganzhou 341000, China; National Navel Orange Engineering Research Center, College of Life Sciences, Gannan Normal University, Ganzhou 341000, China; National Navel Orange Engineering Research Center, College of Life Sciences, Gannan Normal University, Ganzhou 341000, China; National Navel Orange Engineering Research Center, College of Life Sciences, Gannan Normal University, Ganzhou 341000, China; National Navel Orange Engineering Research Center, College of Life Sciences, Gannan Normal University, Ganzhou 341000, China; National Navel Orange Engineering Research Center, College of Life Sciences, Gannan Normal University, Ganzhou 341000, China; National Navel Orange Engineering Research Center, College of Life Sciences, Gannan Normal University, Ganzhou 341000, China; National Navel Orange Engineering Research Center, College of Life Sciences, Gannan Normal University, Ganzhou 341000, China; National Navel Orange Engineering Research Center, College of Life Sciences, Gannan Normal University, Ganzhou 341000, China; Jiangxi Provincial Key Laboratory of Pest and Disease Control of Featured Horticultural Plants, Gannan Normal University, Ganzhou 341000, China; School of Agriculture and Biology, Shanghai Jiao Tong University, Shanghai 200240, China; National Navel Orange Engineering Research Center, College of Life Sciences, Gannan Normal University, Ganzhou 341000, China; Jiangxi Provincial Key Laboratory of Pest and Disease Control of Featured Horticultural Plants, Gannan Normal University, Ganzhou 341000, China

## Abstract

Citrus huanglongbing, caused by *Candidatus* Liberibacter asiaticus (CLas), is associated with severe metabolic disruption, chronic immune activation, and progressive decline of citrus trees, but the posttranslational mechanisms underlying these responses remain poorly understood. Here, we combined ubiquitinated peptide immunoaffinity enrichment with 4D label-free quantitative proteomics to profile protein abundance and protein ubiquitination in infected sweet orange leaves. Infection caused extensive proteome remodeling and a strong global increase in protein ubiquitination. Integrated analysis revealed a predominant group of proteins with reduced abundance and increased ubiquitination, pointing to ubiquitination-associated depletion of proteins involved in chloroplast function, redox homeostasis, and primary metabolism. The degradation machinery itself was also remodeled, including widespread ubiquitination of proteasome subunits and increased proteasome-dependent bulk protein turnover. Physiological and biochemical analyses showed that infection suppressed the hydrogen peroxide-scavenging system, with reduced abundance and increased ubiquitination of catalase, accompanied by elevated hydrogen peroxide accumulation. We also identified actin as a major downstream target of CLas-induced proteostasis remodeling. Actin proteins underwent enhanced ubiquitination and degradation in infected tissues, accompanied by actin filament disruption, and CsACT7 showed 26S proteasome-dependent degradation. Seven CLas-upregulated ubiquitination sites acted cooperatively to promote CsACT7 degradation, as simultaneous substitution of all 7 lysines with alanine strongly stabilized CsACT7 and reduced CLas accumulation in citrus hairy roots. Together, these findings establish ubiquitination-dependent proteome remodeling as a central feature of the citrus response to CLas infection and link ubiquitination-mediated posttranslational regulation to redox imbalance, cytoskeletal disruption, and disease susceptibility.

## Introduction

Citrus huanglongbing (HLB), caused by the unculturable phloem-limited bacterium *Candidatus* Liberibacter asiaticus (CLas), is the most destructive citrus disease worldwide, causing billions of US dollars in annual losses to the citrus industry (Bové 2006; Singerman and Rogers 2020). Transmitted by the Asian citrus psyllid *Diaphorina citri* or grafting, CLas colonizes phloem sieve elements and induces symptoms including root degeneration, blotchy mottling, yellow shoots, and progressive tree decline (Hung et al. 2004; Bové 2006). Current management mainly relies on vector control, infected tree removal, and replanting, but these measures are costly, labor-intensive, and only partially effective in limiting disease spread (Li et al. 2019; Yuan et al. 2021). Thus, a deeper understanding of the molecular basis of HLB pathogenesis is essential for developing durable control strategies.

Emerging evidence suggests HLB is not simply a consequence of bacterial colonization, but also an immune-mediated disorder (Ma et al. 2022b). In sweet orange (*Citrus sinensis*), CLas infection triggers chronic and systemic immune responses in the phloem, including H_2_O_2_ accumulation, callose deposition, and phloem cell death, all of which are closely associated with symptom development (Ma et al. 2022b). Another hallmark of HLB is excessive starch accumulation in leaves, reflecting profound metabolic disruption and impaired source–sink balance (Achor et al. 2010). Despite these advances, the molecular events linking CLas infection to these downstream pathological responses remain poorly understood.

Multi-omics studies in susceptible sweet orange have begun to elucidate host responses to CLas infection at multiple biological scales (Kim et al. 2009; Fan et al. 2011; Martinelli et al. 2016; Franco et al. 2020; Li et al. 2021; Gao et al. 2023) . Transcriptomic analyses have revealed induction of genes associated with starch biosynthesis, defense signaling, and cell wall remodeling (Kim et al. 2009; Fan et al. 2011; Gao et al. 2023). Proteomic studies have shown suppression of amino acid biosynthesis, photosynthetic machinery, and isoflavonoid metabolism, together with activation of proteasome-associated pathways and pathogenesis-related proteins (Martinelli et al. 2016; Li et al. 2021). Phloem-enriched proteomics further identifies CLas-driven metabolic quiescence—downregulation of ribosomal proteins and glycolysis enzymes—coupled with protease induction (Franco et al. 2020). Collectively, these studies support a model in which HLB involves extensive immune and metabolic reprogramming, but they do not fully explain how host protein abundance and function are reshaped during infection.

Posttranslational regulation is central to cellular reprogramming during stress and immunity, and ubiquitination is one of its most versatile mechanisms (Ma et al. 2021). By altering protein stability, activity, localization, and interaction dynamics, ubiquitination can rapidly redirect cellular responses to environmental and biotic challenges (Hu and Sun 2016). In the canonical ubiquitination pathway, ubiquitin is transferred to substrate proteins through the sequential action of E1 activating enzymes, E2 conjugating enzymes, and E3 ligases (Vierstra 2009). Through this E1–E2–E3 cascade, ubiquitination controls receptor trafficking, protein complex assembly, enzymatic activity, and protein degradation, thereby modulating signaling output and cellular homeostasis (Dikic and Schulman 2023).

Most ubiquitinated proteins are ultimately degraded by the 26S proteasome, which consists of the 20S core particle (CP) and 1 or 2 19S regulatory particles (RPs) (Vierstra 2009). The RP recognizes polyubiquitinated substrates, removes ubiquitin chains, unfolds target proteins, and translocates them into the CP for proteolysis. The CP is composed of 4 stacked heptameric rings arranged in an αββα configuration and built from related α (PAA–PAG) and β (PBA–PBG) subunits (Fu et al. 1998a). The RP contains a hexameric ring of ATPases (RPT1–RPT6) together with multiple non-ATPase subunits (RPN1–RPN3, RPN5–RPN13, and RPN15) (Fu et al. 1999; Yang et al. 2004; Lin et al. 2024). In *Arabidopsis*, most CP and RP subunits are encoded by duplicated genes, with several notable exceptions (Lin et al. 2024). Functionally, the RPT ATPases promote substrate unfolding and translocation, whereas RPN10 and RPN13 act as ubiquitin receptors and RPN11 functions as a deubiquitylating enzyme that removes ubiquitin before substrate degradation (Fu et al. 1998b; Husnjak et al. 2008).

In plants, the ubiquitin–proteasome system (UPS) serves as a central regulator of immunity and stress adaptation by dynamically governing the abundance of key signaling proteins (Vierstra 2009). The UPS operates across both hormone-dependent and receptor-mediated immune pathways. For instance, proteasomal degradation of JASMONATE ZIM-DOMAIN (JAZ) repressors activates jasmonate signaling, which is essential for defense against necrotrophic pathogens, whereas ubiquitination-dependent turnover of NONEXPRESSER OF PATHOGENESIS-RELATED GENE 1 (NPR1) fine-tunes salicylic acid (SA)-mediated immunity against biotrophic pathogens (Chini et al. 2007; Thines et al. 2007; Spoel et al. 2009). Ubiquitination also regulates immune receptor abundance and signaling strength: PUB12/13 promotes degradation of FLAGELLIN SENSING 2 (FLS2), SNIPER1/2 controls the stability of nucleotide-binding leucine-rich repeat receptors, and OsCIE1 restrains OsCERK1 activity prior to chitin-triggered activation of immunity (Lu et al. 2011; Wu et al. 2020; Wang et al. 2024).

Recent studies have further linked the UPS to HLB susceptibility. A citrus U-box E3 ligase, PUB21, promotes susceptibility to CLas by ubiquitinating and destabilizing MYC2, whereas stabilizing MYC2 enhances jasmonate-mediated defense and improves resistance to CLas infection (Zhao et al. 2025). Given its central role in defense signaling, proteostasis, and stress adaptation, the UPS is well positioned to connect CLas infection with the physiological and cellular abnormalities characteristic of HLB. However, whether ubiquitination-mediated proteome remodeling broadly contributes to HLB pathogenesis, and which host proteins are targeted, remain largely unknown.

Recent advances in ubiquitinated peptide enrichment and quantitative proteomics have enabled systematic analysis of ubiquitination dynamics during plant–pathogen interactions (Chen et al. 2018; Cheng et al. 2020; Ma et al. 2021; Li et al. 2024). In this study, we used anti-K-ε-GG antibody-based enrichment coupled with 4D label-free quantitative proteomics to profile ubiquitination events in CLas-infected sweet orange (*C. sinensis* cv. Newhall) leaves (Udeshi et al. 2013; Prianichnikov et al. 2020). By integrating ubiquitinome and proteome datasets, we examined how CLas infection remodels host proteostasis and how these changes are linked to downstream physiological dysfunction. Our analyses uncover widespread reprogramming of the citrus UPS, associated with enhanced protein depletion, suppression of antioxidant machinery, and multisite ubiquitination-dependent destabilization of the actin cytoskeleton, providing a mechanistic framework for oxidative stress, cytoskeletal disruption, and disease susceptibility during HLB.

## Results

### Proteome remodeling and ubiquitination dynamics in CLas-infected citrus

**Figure 1:**
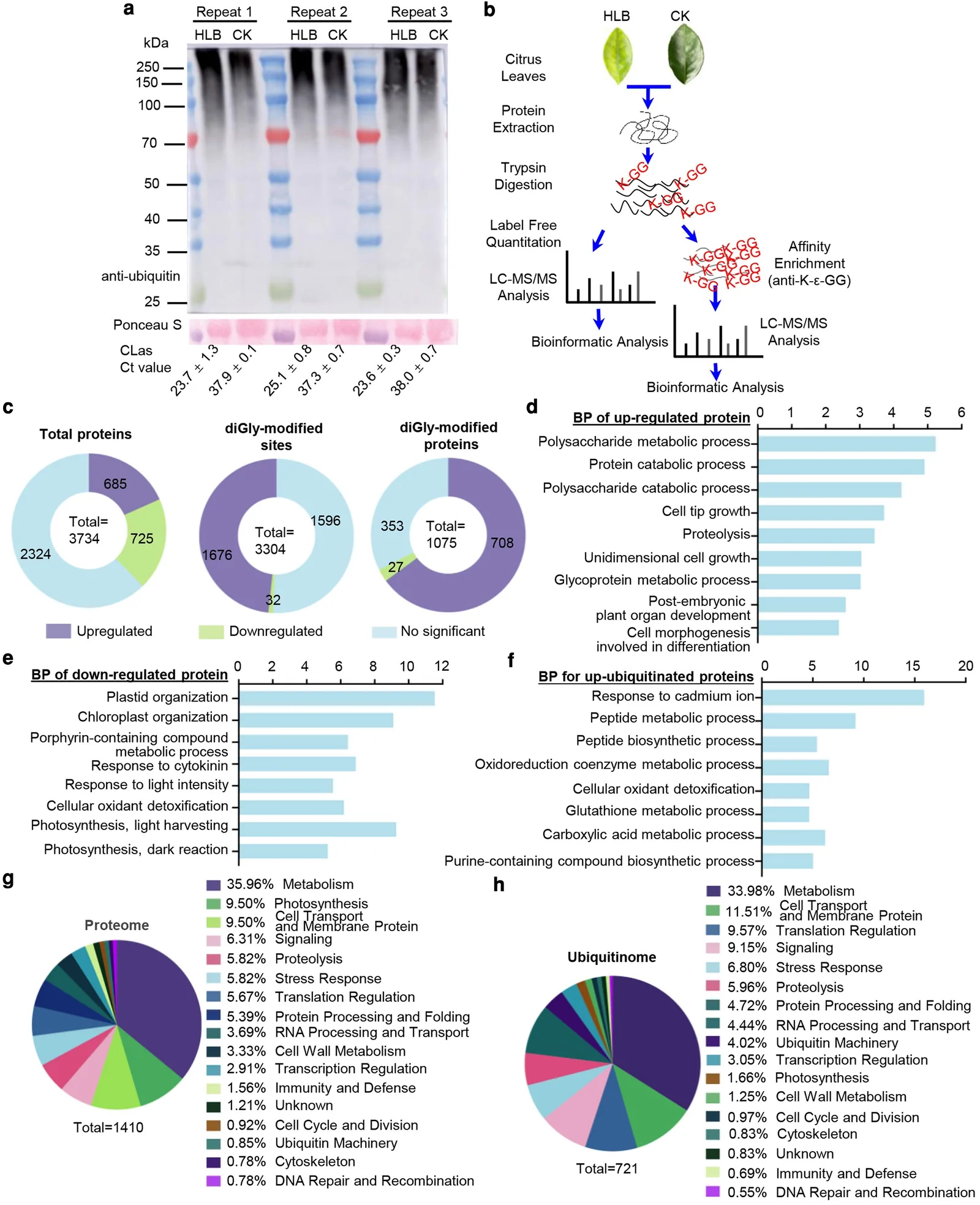
Quantitative proteomic and ubiquitinomic profiling of CLas-infected *C. sinensis* cv. Newhall leaves. a) Immunoblot analysis of total ubiquitinated proteins in leaves from CLas-negative (CK) and CLas-positive (HLB) Newhall trees. Total leaf proteins (30 μg) were probed with an anti-ubiquitin antibody. CLas titers were determined by qPCR using the RNRf/RNRr primer pair targeting the bacterial *ribonucleotide reductase β-subunit* (*RNR*) gene. A Ct value of approximately 36 for the water control indicates that samples with Ct values ≥36 were considered CLas-negative. Lower Ct values indicate higher CLas titers. Ponceau S staining was used as a loading control. Data are shown as mean ± SD (*n* = 3 biologically independent trees). b) Experimental workflow for quantitative proteome and ubiquitinome analyses of citrus leaves. Total proteins extracted from CK and HLB leaves were digested with trypsin. Ubiquitinated peptides were enriched using anti-K-ε-GG antibody-conjugated beads and analyzed by LC–MS/MS. In parallel, 4D label-free quantitative proteomics was performed to normalize ubiquitination changes relative to protein abundance. c) Summary of DEPs, differentially ubiquitinated sites (DUSs), and DUPs identified in the proteome and ubiquitinome datasets. Thirteen DUPs contained both up- and downregulated ubiquitinated peptides. d to f) GO biological process enrichment analysis of upregulated proteins (d), downregulated proteins (e), and proteins with increased ubiquitination (f). Enrichment analysis was performed against the background of all predicted proteins in *Citrus sinensis*. Significance was calculated using Fisher's exact test, and terms with *P* < 0.05 were considered significantly enriched. The *y*-axis indicates GO terms, and the *x*-axis shows −log10(*P-*value). g and h) Functional classification of DEPs (g) and DUPs (h) based on their annotated biological roles.

Immunoblotting with an anti-ubiquitin antibody revealed pronounced accumulation of high-molecular-weight ubiquitin-protein conjugates in CLas-positive (HLB) citrus leaves compared to CLas-negative (CK) controls (Fig. 1a). To characterize these changes, quantitative proteomic and ubiquitinomic analyses were performed (Fig. 1b). Complete datasets of identified peptides, proteins, and modified sites are provided in Dataset S1 (proteome) and Dataset S2 (ubiquitinome). Integrated analysis results revealed extensive CLas-induced proteostasis reprogramming (Fig. 1c). Principal component analysis showed clear separation between CK and HLB groups (Figures S1b and S2b).

Proteome profiling quantified 3,734 proteins (Figure S1a), including 1,410 differentially expressed proteins (DEPs; 685 upregulated, 725 downregulated) (Fig. 1c; Dataset S3), with chloroplasts (50.74%) and cytoplasm (18.94%) as major reservoirs (Figure S1c). DEPs were enriched in metabolic processes (35.96%), photosynthesis (9.50%), cell transport and membrane proteins (9.50%), and signaling (6.31%) (Fig. 1g). Strikingly, Lon protease (92.6-fold) and subtilisin-like protease (34.9-fold) exhibited extreme upregulation, contrasting with the suppression of heat shock proteins (Figure S1d).

Ubiquitinome profiling identified 3,304 quantifiable lysine ubiquitination (Kub) sites, including 1,708 differentially modified sites (DUS, 1,676 upregulated, 32 downregulated) (Fig. 1c; Dataset S4). In total, 735 differentially ubiquitinated proteins (DUPs) were detected (708 upregulated and 27 downregulated), including 13 proteins that contained both increased and decreased Kub sites (Fig. 1c; Dataset S5). DUPs were enriched in metabolic processes (33.98%), cell transport and membrane proteins (11.51%), translation regulation (9.57%), and signaling (9.15%) (Fig. 1h; Dataset S5) and localized primarily to cytoplasm (42.52%) and chloroplasts (23.41%) (Figure S2c). Analysis of Kub site distribution revealed most DUPs (68.51%) contained 1 to 2 modification sites (Figure S2d; Dataset S6), while hyperubiquitinated proteins (≥7 sites) were predominantly heat shock proteins, metabolic enzymes, oxidative stress responders, and cytoskeletal/signaling components (Figure S3a). Notably, hyperubiquitination of ubiquitin machinery components—ubiquitin, E1-activating enzyme, and ubiquitin hydrolase—highlighted UPS self-regulation (Figure S3a).

Gene Ontology (GO) enrichment analyses revealed upregulated DEPs in polysaccharides and proteins catabolic processes versus downregulated DEPs in photosynthesis, oxidant detoxification, and cytokinin responses, while upregulated DUPs were linked to cadmium ion response, peptide metabolism, and oxidant detoxification (Fig. 1, d to f; Figures S1, e to h, and S3, b to e; Datasets S7 and S8). Analysis of the top 15 up- and downregulated proteins showed increased abundance of lipoxygenase, β-1,3-glucanase, subtilases, and dehydrin indicated activation of defense/stress responses, whereas decreased chlorophyll a/b-binding proteins and fructose-bisphosphate aldolase reflected repression of photosynthetic and carbon metabolism pathways (Figure S1d).

### Proteome–ubiquitinome integration reveals ubiquitination-linked depletion of chloroplast and metabolic proteins during HLB

**Figure 2:**
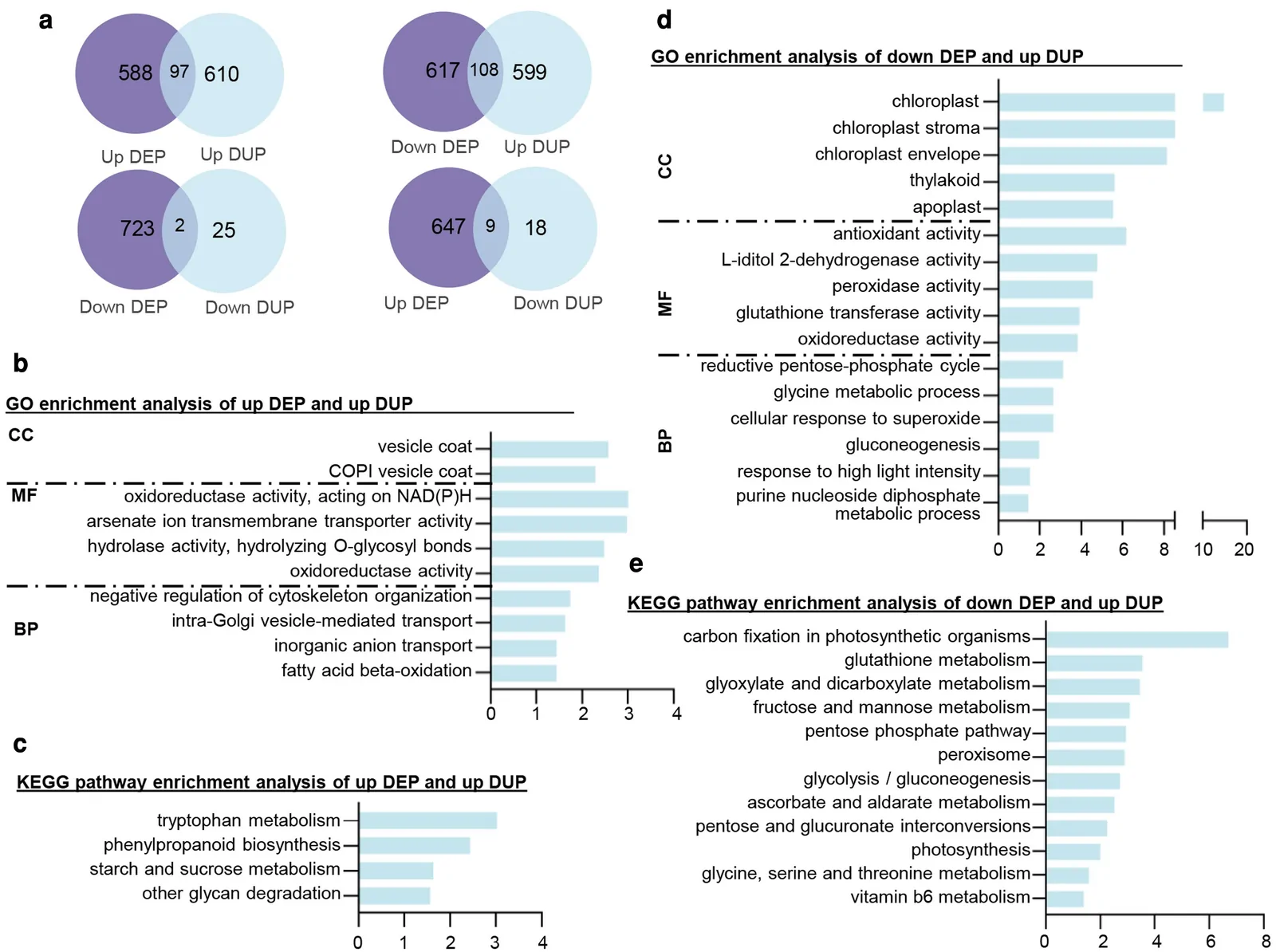
Coordinated changes in protein abundance and ubiquitination in CLas-infected citrus leaves. a) Venn diagrams showing the overlap among proteins exhibiting 4 combined patterns of change in the proteome and ubiquitinome: increased protein abundance (Up DEP) with increased ubiquitination (Up DUP), decreased abundance (Down DEP) with increased ubiquitination (Up DUP), decreased abundance (Down DEP) with decreased ubiquitination (Down DUP), and increased abundance (Up DEP) with decreased ubiquitination (Down DUP). b and c) GO term enrichment (b) and KEGG pathway enrichment (c) analyses of proteins that were significantly increased at both the protein abundance level and the ubiquitination level. d and e) GO term enrichment (d) and KEGG pathway enrichment (e) analyses of proteins with decreased abundance but increased ubiquitination.

To assess how ubiquitination contributes to proteome remodeling during HLB, we integrated the proteome and ubiquitinome datasets. Four major patterns of protein abundance and ubiquitination were identified (Fig. 2a). Ninety-seven proteins showed coordinated increases in abundance and ubiquitination, whereas only 2 showed coordinated decreases. By contrast, 117 proteins displayed inverse trends, including 108 with decreased abundance but increased ubiquitination and 9 with the opposite pattern (Fig. 2a; Dataset S9). The predominance of proteins with reduced abundance and elevated ubiquitination is consistent with UPS-mediated protein turnover in plant–microbe interactions (Liu et al. 2024).

Proteins with increased abundance and ubiquitination were enriched in COPI vesicle coat components, oxidoreductase and hydrolase activities, intra-Golgi vesicle-mediated transport, fatty acid β-oxidation, and negative regulation of cytoskeleton organization (Fig. 2b; Dataset S10). Kyoto Encyclopedia of Genes and Genomes (KEGG) analysis further highlighted tryptophan metabolism, phenylpropanoid biosynthesis, and starch and sucrose metabolism (Fig. 2c; Dataset S10), indicating activation of stress-related metabolism, vesicle trafficking, and carbohydrate reprogramming.

In contrast, proteins with decreased abundance but increased ubiquitination were strongly enriched in chloroplast-associated functions, antioxidant activity, the reductive pentose phosphate cycle, glycine metabolism, and cellular responses to superoxide (Fig. 2d; Dataset S11). KEGG analysis identified photosynthesis, carbon fixation, glutathione metabolism, the pentose phosphate pathway, glycolysis/gluconeogenesis, peroxisome, ascorbate and aldarate metabolism, and related carbon metabolic pathways (Fig. 2e; Dataset S11). These data indicate that chloroplast function, redox homeostasis, and primary metabolism are major targets of ubiquitination-linked protein depletion during HLB.

### Multi-omics integration uncovers transcriptional and posttranscriptional regulation of CLas-responsive pathways

To further define the regulatory basis of these changes, we integrated the proteome and ubiquitinome datasets with a previously reported transcriptome dataset (Li et al. 2024). This analysis identified 73 genes with coordinated changes at the transcript, protein, and ubiquitination levels (Dataset S12).

One major group, including photosynthetic, redox, and metabolic proteins such as PSI reaction center subunit II, thioredoxin-dependent peroxiredoxin, and fructose-1,6-bisphosphatase, showed reduced transcript abundance, reduced protein abundance, and increased ubiquitination (Dataset S12), consistent with transcriptional repression coupled with UPS-associated protein clearance. A second group, including caffeic acid *O*-methyltransferases, ABC transporters, adenosine kinase, and an E3 ligase, showed increased transcript abundance, increased protein abundance, and increased ubiquitination (Dataset S12), suggesting that ubiquitination in this group may serve non-proteolytic roles in stress signaling or adaptive responses.

Notably, 8 genes, including ascorbate peroxidase 2 (CsAPX2), cinnamoyl-CoA reductase, and a small heat shock protein, were transcriptionally induced but reduced at the protein level (Dataset S12), highlighting the importance of posttranscriptional regulation. Together, these results indicate that the citrus response to CLas infection is shaped by both transcriptional reprogramming and UPS-dependent protein turnover.

### Comparative analysis of Kub site architecture in citrus leaves

**Figure 3:**
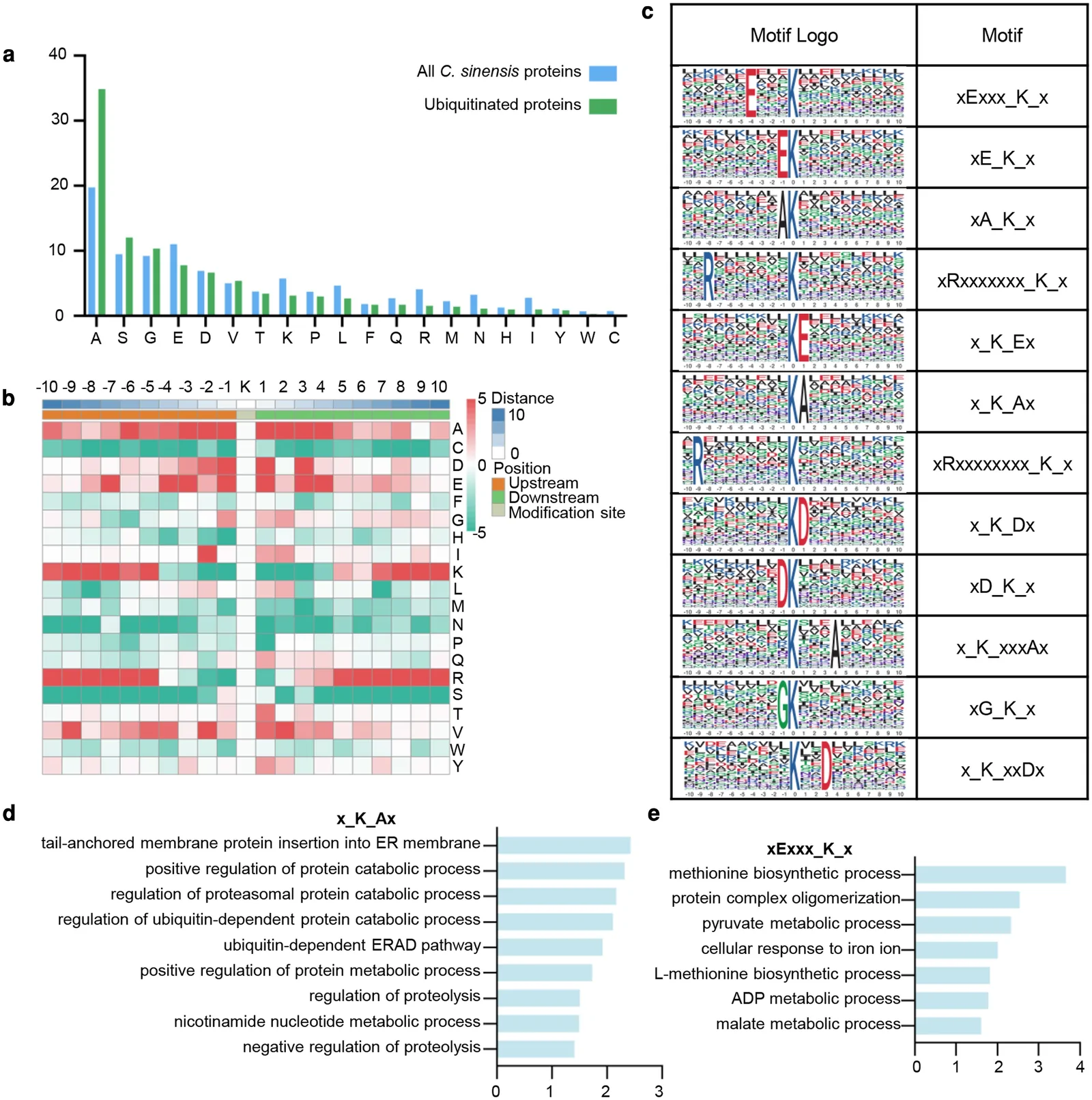
The amino acid environment surrounding ubiquitinated lysine residues. a) A histogram comparing the frequencies of amino acids immediately following the N-terminal methionine in DUPs (green) versus all *C. sinensis* proteins (blue). b) Heatmap illustrates the relative abundance of amino acids surrounding ubiquitinated lysine residues compared to all potential modification sites in *C. sinensis*. Red indicates enrichment, while green indicates depletion. The analysis was conducted using the MoMo tool with the motif-x algorithm on peptide segments spanning 10 amino acids upstream and downstream of ubiquitinated lysines. A motif was defined when the peptide count exceeded 20, and the statistical test yielded a *P*-value < 0.000001. c) Consensus sequence logo depicting the relative sizes of amino acid residues around ubiquitinated lysine residues, proportional to their abundance across all analyzed peptides. The *x*-axis lists residues flanking the ubiquitinated lysine. Residue colors reflect side-chain chemistry: green for polar uncharged, purple for polar amide, blue for basic, red for acidic, and black for hydrophobic residues. d and e) GO enrichment of DUPs containing the x_K_Ax motif (d) and xExxx_K_x (e) in biological processes. Analyses were conducted against the proteins identified in the proteome. Fisher's exact test was utilized to compute *P-*values, with *P* < 0.05 deemed statistically significant. The vertical axis indicates the biological processes, while the horizontal axis represents the -log10(*P-*values).

To identify sequence features associated with Kub in citrus, we analyzed the N-terminal properties of DUPs and the amino acid composition surrounding Kub sites in *C. sinensis* leaves. Ala, Ser, and Gly were more frequent immediately after the initiator methionine in DUPs than in the global citrus proteome (Fig. 3a), suggesting that differential ubiquitination during CLas infection is associated with N termini favorable for initiator methionine removal and protein turnover (Gibbs et al. 2014). Analysis of residues flanking Kub sites showed that Ala, Asp, Glu, Gly, and Val were enriched near modified lysines, whereas Lys and Arg were depleted in the immediate vicinity but increased at more distal positions (Fig. 3b; Dataset S13). This pattern closely resembled that reported for the citrus fruit ubiquitinome (Cheng et al. 2020), indicating that local Kub site architecture is conserved between citrus leaves and fruits.

Motif analysis identified 12 Kub motifs in citrus leaves (Fig. 3c; Dataset S13), including 3 also detected in citrus fruit (xExxx_K_x, xE_K_x, and xD_K_x) (Cheng et al. 2020). Functional enrichment of motif-containing DUPs revealed clear motif-specific associations (Fig. 3, d and e; Figure S4, a to j; Dataset S14). Proteins carrying the x_K_Ax motif were enriched in nicotinamide nucleotide metabolism, ER protein quality control, and protein turnover (including tail-anchored protein insertion, proteasome/ubiquitin-dependent catabolism, ERAD, and proteolysis regulation) (Fig. 3d). In contrast, proteins containing the xExxx_K_x motif were enriched mainly in metabolic functions, including methionine biosynthesis, pyruvate and malate metabolism, ADP metabolism, and iron-responsive processes (Fig. 3e). Other motifs were associated with additional functions related to carbohydrate and nucleotide metabolism, proteolysis, and stress responses (Figure S4, a to j; Dataset S14). Together, these results indicate that distinct Kub motifs are associated with functionally distinct classes of ubiquitinated proteins in citrus leaves.

Notably, H_2_O_2_-scavenging enzymes were most frequently associated with the xE_K_x and x_K_Ax motifs, whereas ubiquitinated actin peptides were enriched mainly in the x_K_Dx motif class (Dataset S15). Although the functional significance of these motif associations remains to be tested, DUPs carrying the xE_K_x motif were enriched in ADP metabolic processes, whereas those carrying the x_K_Dx motif were enriched primarily in nucleoside diphosphate phosphorylation-related processes (Figure S4a).

### CLas infection remodels the citrus ubiquitin–proteasome pathway

To test whether CLas affects the degradation machinery itself, we examined ubiquitination of proteasome components and other ubiquitin-system factors in *C. sinensis*. Comparative analysis revealed both conservation and diversification of the citrus proteasome relative to *A. thaliana*: *C. sinensis* lacks clear homologs of *AtRPN15* but retains duplicated paralogs encoding several 20S core subunits (PAA, PAC, PAD, PAE, PBB, and PBF) and 19S RP components (RPT1/RPT2 and RPN3/5/6/7/10) (Figures S5 and S6) (Book et al. 2010).

**Figure 4:**
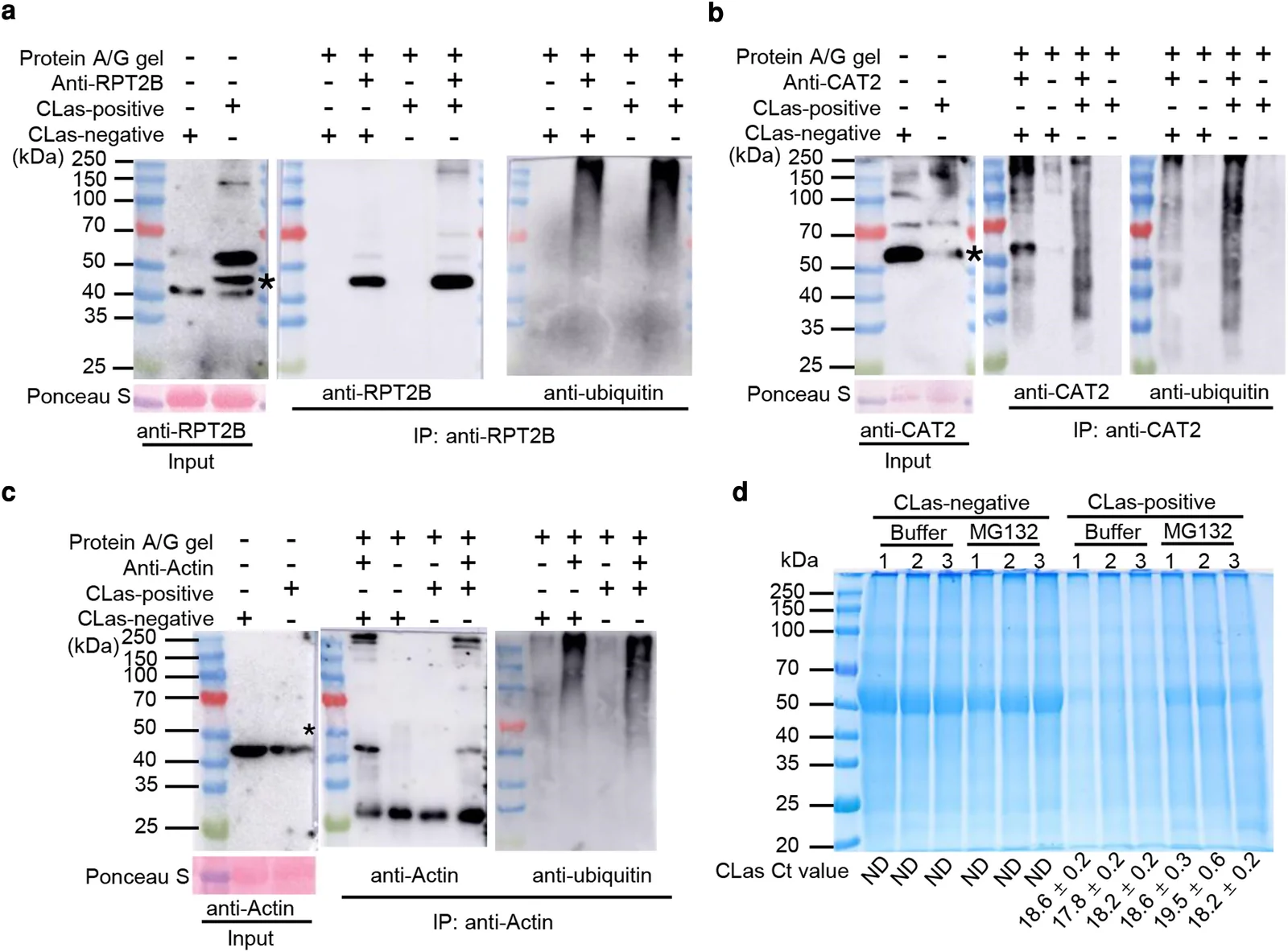
In vivo validation of candidate ubiquitination and proteasome-dependent protein turnover in CLas-infected newhall leaves. a to c) Ubiquitination of CsRPT2B (a), CsCatalase (b), and CsActin (c) in CLas-negative and CLas-positive leaves. Total proteins (1 mg) from CLas-negative and CLas-positive Newhall leaves were immunoprecipitated with the indicated specific antibodies crosslinked with Protein A/G beads; the eluted proteins were immunoblotted with the corresponding antibodies and an anti-ubiquitin antibody. d) Proteasome inhibition promotes protein accumulation in CLas-positive leaves. CLas-negative and CLas-positive leaves were infiltrated with buffer (10 mM MgCl_2_ and 10 mM MES-KOH [pH 5.6 to 5.8]) or buffer containing 50 μM MG132. Leaf samples were harvested 24 h after treatment. Total proteins extracted from 0.1 g fresh tissue were separated by SDS-PAGE and stained with Coomassie Brilliant Blue. Three biological replicates were included per treatment.

CLas infection triggered ubiquitination of 50% of 20S core protease subunits (10 out of 20), including key structural components such as CsPAA1, CsPAA2, CsPAC2, CsPAE1, CsPAF1, CsPAG1, CsPBB1, CsPBD1, CsPBE1, and CsPBG1, and 44% of 19S RP subunits (11 out of 25), comprising both ATPase (CsRPT1A/2B/4/6) and non-ATPase components (CsRPN2/3B/7A/7B/8/10A/10B) (Table 1). Except for CsRPN10B-K57, all identified Kub sites in 26S proteasome subunits were significantly upregulated in infected leaves, indicating extensive but subunit-specific proteasome remodeling (Table 1). Consistently, anti-CsRPT2B immunoprecipitation followed by anti-ubiquitin immunoblotting detected ubiquitinated CsRPT2B in both CLas-negative and CLas-positive leaves, with increased CsRPT2B abundance in HLB leaves (Fig. 4a).

**Table 1:** Ubiquitination profiling of 26S proteasome subunits in CLas-infected citrus.

Gene	Protein accession	Modification sites (fold change)						
20S core protease (CP)								
CsPAA1	Cs_ont_1g019080.1	K28 (1.825)	K40 (2.319)	K217 (2.961)	K30 (2.948)			
CsPAA2	Cs_ont_4g022110.1	K30 (2.948)						
CsPAB1	Cs_ont_9g028000.1							
CsPAC1	Cs_ont_2g010310.1							
CsPAC2	Cs_ont_6g011410.1	K54 (1.686)	K176 (2.350)	K235 (1.622)	K239 (1.660)			
CsPAD1	Cs_ont_1g028410.1							
CsPAD2	Cs_ont_8g027790.1							
CsPAE1	Cs_ont_1g018210.1	K520 (1.620)	K522 (3.148)					
CsPAE2	Cs_ont_4g020700.1							
CsPAF1	Cs_ont_3g003410.1	K41 (4.900)	K169 (1.687)					
CsPAG1	Cs_ont_6g006570.1	K43 (3.412)	K57 (2.022)	K93 (1.830)	K180 (2.279)			
CsPBA1	Cs_ont_4g000250.1							
CsPBB1	Cs_ont_2g027880.1	K68 (2.539)	K247 (2.491)	K254 (3.319)				
CsPBB2	Cs_ont_7g023430.2							
CsPBC1	Cs_ont_3g009020.1							
CsPBD1	Cs_ont_9g005970.1	K197 (1.992)						
CsPBE1	Cs_ont_7g022440.1	K117 (1.642)						
CsPBF1	Cs_ont_5g001880.2							
CsPBF2	Cs_ont_7g012560.2							
CsPBG1	Cs_ont_8g004500.1	K184 (2.076)	K188 (1.707)	K208 (1.987)				
19S regulatory particle (RP)								
CsRPT1A	Cs_ont_1g011050.2	K39 (1.554)	K40 (1.658)	K121 (1.598)	K179 (1.740)	K349 (2.769)	K400 (2.331)	K421 (1.607)
CsRPT1B	Cs_ont_1g018030.1							
CsRPT2A	Cs_ont_2g001300.1							
CsRPT2B	Cs_ont_5g013130.1	K29 (2.010)	K63 (1.606)	K75 (1.614)	K271 (2.129)	K419 (2.040)		
CsRPT3	Cs_ont_1g006900.1							
CsRPT4	Cs_ont_3g003160.1	K77 (2.416)						
CsRPT5	Cs_ont_3g015350.1							
CsRPT6	Cs_ont_4g006710.1	K23 (1.762)						
CsRPN1	Cs_ont_2g027620.1							
CsRPN2	Cs_ont_9g013220.1	K186 (2.178)	K208 (2.246)	K842 (2.471)	K853 (1.756)	K903 (1.908)	K925 (1.741)	K928 (2.031)
CsRPN3A	Cs_ont_8g028060.1							
CsRPN3B	Cs_ont_2g023700.1	K86 (2.324)	K166 (2.585)	K347 (1.717)	K384 (2.184)	K504 (1.631)	K590 (1.823)	
CsRPN5A	Cs_ont_1g003790.1							
CsRPN5B	Cs_ont_3g003880.1							
CsRPN6A	Cs_ont_4g025300.1							
CsRPN6B	Cs_ont_2g029980.1							
CsRPN7A	Cs_ont_5g044350.1	K16 (1.953)						
CsRPN7B	Cs_ont_5g002560.1	K135 (1.619)						
CsRPN8	Cs_ont_6g015330.1	K207 (1.856)						
CsRPN9	Cs_ont_3g021540.1							
CsRPN10A	Cs_ont_1g028330.1	K57 (1.749)	K123 (1.837)					
CsRPN10B	Cs_ont_8g027720.1	K57 (0.663)						
CsRPN11	Cs_ont_3g004820.1							
CsRPN12	Cs_ont_5g049800.1							
CsRPN13	Cs_ont_4g024820.1							

We next assessed proteolytic output. Using equal fresh weights of Newhall leaves, CLas-positive samples yielded less total protein than CLas-negative controls. This reduction was suppressed by 26S proteasome inhibitor MG132 (Fig. 4d), indicating enhanced proteasome-dependent degradation at the tissue level. Thus, CLas remodels both proteasome ubiquitination and its contribution to bulk proteome turnover.

Beyond the proteasome, ubiquitinome profiling identified 29 ubiquitin-system components, including 1 CsE1, 3 CsE2, 5 CsE3, 2 DUBs, and 6 ubiquitin carboxyl-terminal hydrolase predicted to release free ubiquitin monomers from polyubiquitin chains (Table 2). Notably, the NBR1 ortholog (neighbor of BRCA1, Cs_ont_5g045120.1), a selective autophagy receptor, exhibited both increased and decreased ubiquitination sites after infection (Table 2). Because NBR1 recognizes ubiquitinated cargo and delivers it to autophagosomes via ATG8 interactions (Lamark et al. 2009; Svenning et al. 2011; Jung et al. 2020), these data suggest coordination between the UPS and autophagy during CLas-induced proteostasis remodeling.

**Table 2:** Ubiquitin system components with altered ubiquitination sites in CLas-infected citrus leaves.

Protein accession	Protein description	No. of modified sites	Regulated type
Cs_ont_3g007450.1	Deubiquitinating enzyme MINDY-3	1	Up
Cs_ont_4g018640.2	Deubiquitinating enzyme OTU1	2	Up
Cs_ont_8g000140.2	E1 ubiquitin-activating enzyme	10	Up/Down
Cs_ont_2g026600.1	E2 ubiquitin-conjugating enzyme	2	Up
Cs_ont_7g013090.1	E2 ubiquitin-conjugating enzyme	2	Up
Cs_ont_8g002980.3	E2 ubiquitin-conjugating enzyme	1	Up
Cs_ont_2g011110.1	E3 ubiquitin ligase	3	Up
Cs_ont_3g005900.1	E3 ubiquitin ligase RNF170 like protein	1	Up
Cs_ont_9g028480.1	E3 ubiquitin-protein ligase RGLG4 like protein	1	Up
Cs_ont_3g012230.2	HECT-type E3 ubiquitin ligase	1	Down
Cs_ont_5g028450.1	RING-type E3 ubiquitin ligase	1	Up
Cs_ont_1g029260.3	Ubiquitin-like protein	2	Up
Cs_ont_6g008710.4	Ubiquitin	2	Up
Cs_ont_8g005810.1	Ubiquitin	8	Up
Cs_ont_6g010040.1	Ubiquitin carboxyl-terminal hydrolase	7	Up
Cs_ont_8g009770.1	Ubiquitin carboxyl-terminal hydrolase	10	Up
Cs_ont_1g020540.1	Ubiquitin carboxyl-terminal hydrolase	1	Up
Cs_ont_4g015830.1	Ubiquitin carboxyl-terminal hydrolase	6	Up
Cs_ont_5g042900.1	Ubiquitin carboxyl-terminal hydrolase	3	Up
Cs_ont_6g026660.2	Ubiquitin carboxyl-terminal hydrolase	1	Up
Cs_ont_6g009130.1	Ubiquitin conjugating Enzyme variant	3	Up
Cs_ont_5g034660.1	Ubiquitin-like protein	3	Up
Cs_ont_8g020130.2	Ubiquitin fusion degradation protein 1-like isoform X2	4	Up
Cs_ont_6g003930.1	Ubiquitin receptor RAD23 like protein	4	Up
Cs_ont_7g019460.1	Ubiquitin receptor RAD23 like protein	2	Up
Cs_ont_9g014370.1	Ubiquitin-associated domain-containing protein	3	Up
Cs_ont_5g045120.1	Ubiquitin-associated protein NBR1 like protein	5	Up/Down
Cs_ont_3g001410.1	Ubiquitin-like protein	2	Up
Cs_ont_3g028540.1	Ubiquitin-like protein	1	Up

Together, these findings indicate that CLas infection broadly remodels the host UPS and associated proteostasis pathways, and that this remodeling is accompanied by enhanced proteasome-dependent proteome depletion in infected leaves.

### CLas infection suppresses antioxidant capacity and elevates H_2_O_2_ accumulation

Because our integrated proteome–ubiquitinome analysis showed that proteins with decreased abundance but increased ubiquitination were enriched in antioxidant functions, we next tested whether CLas infection disrupts H_2_O_2_-scavenging capacity in citrus (Fig. 2d). Conserved antioxidant enzymes—catalase (CAT), APX, peroxiredoxins (Prx), glutathione peroxidase-like enzymes (GPXL), and type III peroxidases (CsPRXs)—were identified through genome-wide homology analysis and phylogenetic classification (Figures S7, a to d, and S8; Dataset S16) (Attacha et al. 2017; Liebthal et al. 2018; Smirnoff and Arnaud 2019).

**Figure 5:**
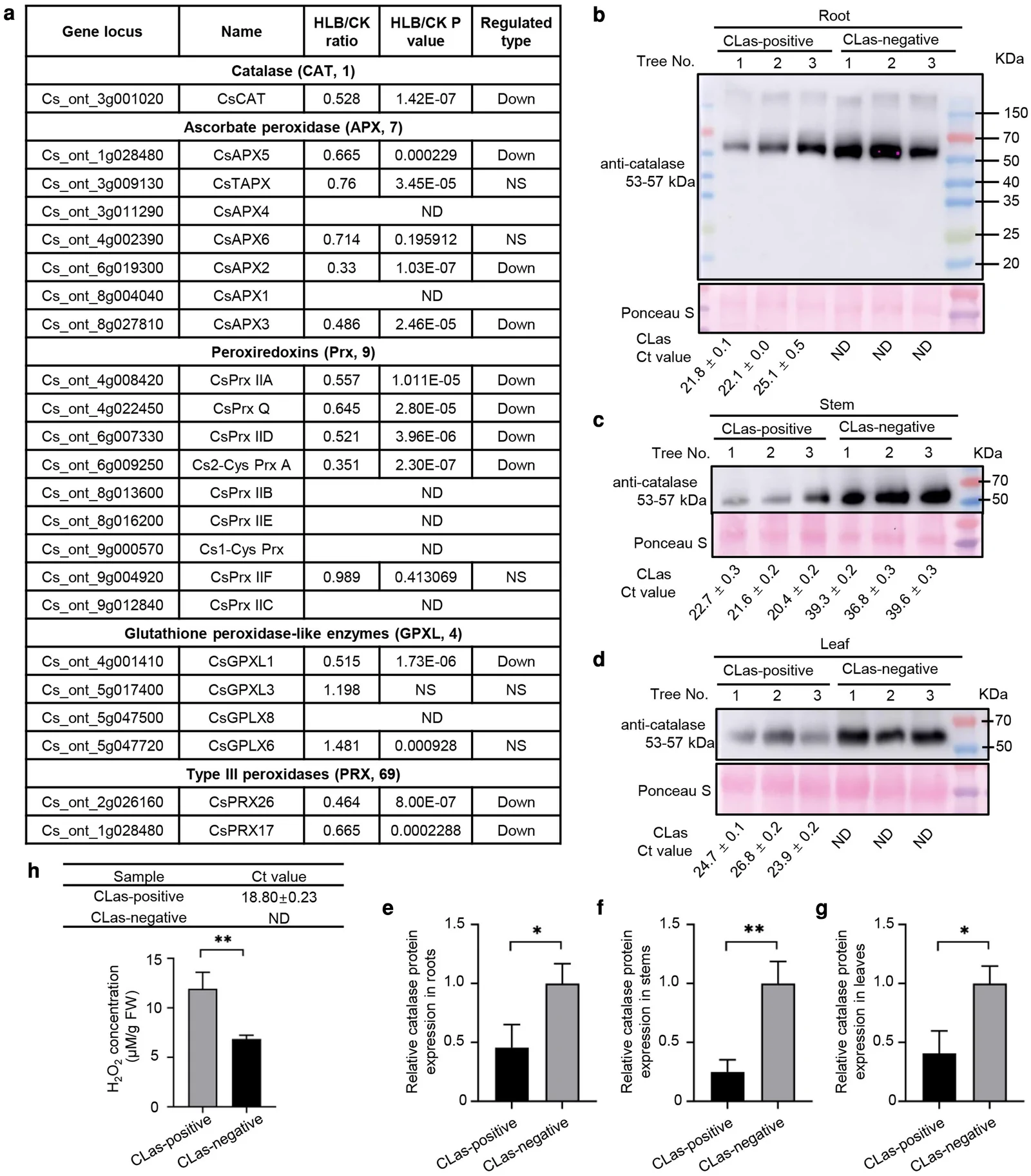
CLas infection suppresses H_2_O_2_-scavenging enzymes in citrus. (a) Proteomic quantification of H_2_O_2_-scavenging enzymes. Plant H_2_O_2_ detoxification systems include hem peroxidases (CAT, APX, PRXs) and thiol-based peroxidases (Prxs, GPXL). CAT: catalase; APX: ascorbate peroxidase; PRXs: type III peroxidases; Prxs: peroxiredoxins; and GPXL: glutathione peroxidase-like enzymes. *C. sinensis* genes encoding these enzymes were identified through phylogenetic analysis of *A. thaliana* H_2_O_2_-detoxification homologs using protein BLAST (Figures S7 and S8). “Up”/“Down”: HLB/CK ratio >1.5/<0.67; “NS”: no significant change; “ND”: not detected. b to d) Immunoblot validation of catalase degradation in roots (b), stems (c), and leaves (d) from CLas-positive and CLas-negative trees. Three CLas-negative and CLas-positive trees were selected for experiments. e to g) Quantification of catalase protein levels in roots (e), stems (f), and leaves (g). Data in (b to g) are presented as mean ± SD, and differences were analyzed using unpaired *t*-tests in GraphPad Prism (*n* = 3 biological replicates; for CLas detection, *n* = 3 technical replicates), *P < 0.05, **P < 0.01. Anti-catalase antibody (PhytoAB, PHY3044A); Ponceau S confirms loading. The WB results were quantified using ImageJ software. CLas titers were validated by qPCR (lower Ct values indicate higher bacterial titers). Ct value ≥ 40 labeled with ND (not detected). h) Determination of H_2_O_2_ concentration in CLas negative or positive leaves using TCA method. Mean and SD (*n* = 3) are shown.

Proteomic profiling revealed that, aside from nonsignificant changes (NS) and upregulation of 7/69 CsPRXs, most detected H_2_O_2_ scavengers were downregulated, including CsCAT, CsAPX2/3/5, CsPrx Q/IIA/IID/IIF/2-CysPrx A, CsGPXL1, and CsPRX17/26, indicating broad impairment of the H_2_O_2_ detoxification system (Fig. 5a; Dataset S16). Immunoblotting with a catalase-specific antibody further showed markedly reduced catalase protein abundance in CLas-infected citrus roots, stems, and leaves (Fig. 5, b to g), suggesting that compromised catalase accumulation is a systemic feature of infection. Consistent with this reduction, TCA-based assays detected significantly higher H_2_O_2_ levels in CLas-positive leaves than in CLas-negative controls (Fig. 5h).

Ubiquitinome analysis further showed that CsCAT carried increased ubiquitination at 8 lysine residues, whereas CsAPX2 and CsAPX3 displayed elevated ubiquitination at 3 and 1 lysine residues, respectively (Figure S7, e and f). Consistently, immunoprecipitation with an anti-CAT2 antibody followed by anti-ubiquitin immunoblotting detected ubiquitinated catalase in both CLas-negative and CLas-positive leaves, with a much stronger signal in infected samples (Fig. 4b). Together with the reduced catalase protein abundance in infected tissues, these results indicate that catalase and APX isoforms are ubiquitinated components of the citrus H_2_O_2_-scavenging system and support a model in which CLas infection suppresses antioxidant capacity, thereby exacerbating oxidative stress.

Taken together, these findings provide physiological support for the redox-related signatures identified by multi-omics analysis and position antioxidant suppression as a major downstream outcome of CLas-associated UPS remodeling.

### CLas infection promotes UPS-dependent actin degradation and cytoskeletal disruption

To test whether CLas-induced UPS remodeling affects specific downstream processes, we examined the cytoskeleton, which was highlighted by the integrated proteome–ubiquitinome analysis. Proteins involved in negative regulation of cytoskeleton organization showed coordinated increases in abundance and ubiquitination in infected leaves (Fig. 2b), indicating cytoskeletal remodeling as a prominent outcome of infection.

Ubiquitinome profiling identified 6 cytoskeletal proteins with elevated ubiquitination during CLas infection, including actin isoforms CsACT7 and CsACT4 (Table 3; Figure S9, a and b). Phylogenetic analysis grouped *C. sinensis* actins into vegetative (*CsACT7/8*; clustering with *Arabidopsis AtACT2/7/8*) and reproductive (*CsACT3/4/5/6*; *AtACT1/3/4/11/12*) clades (Figure S9a) (Meagher et al. 1999). However, semiquantitative PCR showed broad tissue expression of all *CsACT* genes (Figure S9c), indicating multiple isoforms function in vegetative tissues.

**Table 3:** Cytoskeleton proteins with elevated ubiquitination in CLas-infected citrus.

Protein accession	Position	HLB/CK ratio	HLB/CK *P-*value	Protein description	Modified sequence
Cs_ont_1g004160.1	K52	1.902	0.0016	Actin	HTGVMVGMGQK(1)DAYVGDEAQSKR
	K63	2.236	0.0034		HTGVMVGMGQK(1)DAYVGDEAQSK(1)R
	K115	1.73	0.0005		VAPEEHPVLLTEAPLNPK(1)ANR
	K233	1.806	0.0086		LAYVALDYEQELETAK(1)SSSSVEK
	K240	1.503	0.0157		SSSSVEK(1)NYELPDGQIITIGAER
	K328	2.45	0.0001		EITALAPSSMK(1)IK(1)VVAPPER
	K330	2.014	0.0003		IK(1)VVAPPER
Cs_ont_1g022110.1	K132	1.684	0.0047	Kinesin	ISPLINEK(0.816)K(0.184)
	K718	2.166	0.0009		TEGTVALVK(1)SSSEK
	K1103	1.818	0.0087		K(1)AMLTSLDELAER
Cs_ont_6g017570.1	K328	2.368	0.0008	Actin	EISALAPSSMK(0.976)IK(0.024)
	K330	2.785	7E-05		IK(1)VVAPPER
Cs_ont_7g004070.1	K22	3.762	0.0154	Actin depolymerizing factor	FLELK(0.977)AK(0.023)
	K96	4.147	8E-05		IIFIAWSPDTSK(1)VR
Cs_ont_8g003160.1	K69	3.412	0.0028	Actin depolymerizing factor	IEEK(1)QK(1)QVIVEK
	K71	3.412	0.0028		IEEK(1)QK(1)QVIVEK
Cs_ont_6g017040.1	K3	1.733	0.001	TOG domain-containing protein	AK(1)DGPNWDGLLK
	K199	3.558	0.0032		DALGSESVK(1)FQR
	K161	1.968	0.0095		TK(1)ALGAISSLIR

The red “K” denotes ubiquitination modification sites, and the numbers in parentheses represent the proportion of ubiquitination modifications.

**Figure 6:**
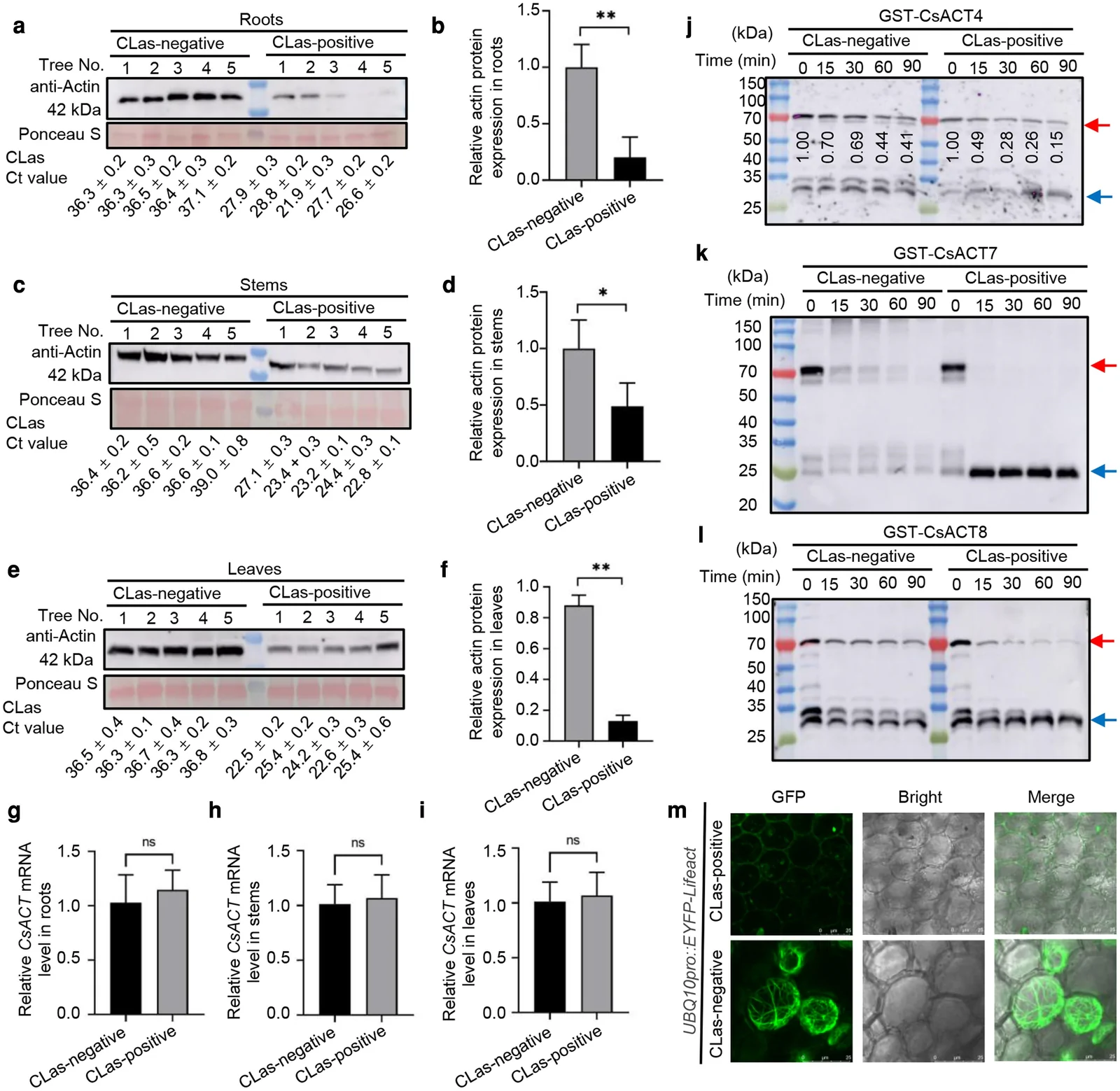
CLas infection promotes actin cytoskeleton degradation in citrus tissues. a to f) Immunoblot analysis of actin protein abundance in roots (a and b), stems (c and d), and leaves (e and f) collected from CLas-positive and CLas-negative Newhall trees (*n* = 5 biological tree replicates). Immunoblot signals were quantified using ImageJ software. An anti-Actin antibody (PTM BIO, PTM-6702) was used for detection, and Ponceau S staining was used as a loading control. g to i) RT-qPCR analysis of *CsACT* transcript levels in the corresponding roots (g), stems (h), and leaves (i). Data in (a to i) are presented as mean ± SD. Statistical significance was determined using unpaired 2-tailed *t*-tests in GraphPad Prism (*n* = 5 biological replicates; CLas detection *n* = 3 technical replicates), *P < 0.05, **P < 0.01. (j to l) In vitro degradation assays of recombinant GST-CsACT4 (j), GST-CsACT7 (k), and GST-CsACT8 (l). Purified recombinant proteins were incubated with protein extracts from CLas-positive or CLas-negative leaves at 25 °C for the indicated time. Protein abundance was monitored by immunoblotting with an anti-GST antibody. Experiments were repeated at least twice with similar results. Blue arrow: GST; Red arrow: GST-CsACT proteins. m) Confocal imaging of F-actin networks in *Agrobacterium rhizogenes* (K599)-transformed *Citrus trifoliata* roots expressing UBQ10pro::EYFP*-Lifeact*. CLas titers were validated by qPCR for each sample.

Immunoblotting with a universal plant anti-actin antibody revealed actin degradation in roots (Fig. 6, a and b), stems (Fig. 6, c and d), and leaves (Fig. 6, e and f) after CLas infection, whereas *CsACT* transcript levels were unchanged (Fig. 6, g to i), indicating posttranscriptional depletion. Recombinant GST-CsACT4/7/8 proteins were purified (Figure S10, i and j) and incubated with leaf extracts; extracts from infected leaves accelerated degradation of all 3 proteins, with GST-CsACT7 most unstable (Fig. 6, j to l). Confocal imaging of transgenic hairy roots expressing UBQ10pro::EYFP-Lifeact confirmed actin filament disruption in infected tissues (Fig. 6m; Figure S11). Thus, CLas infection promotes actin degradation and microfilament disassembly.

**Figure 7:**
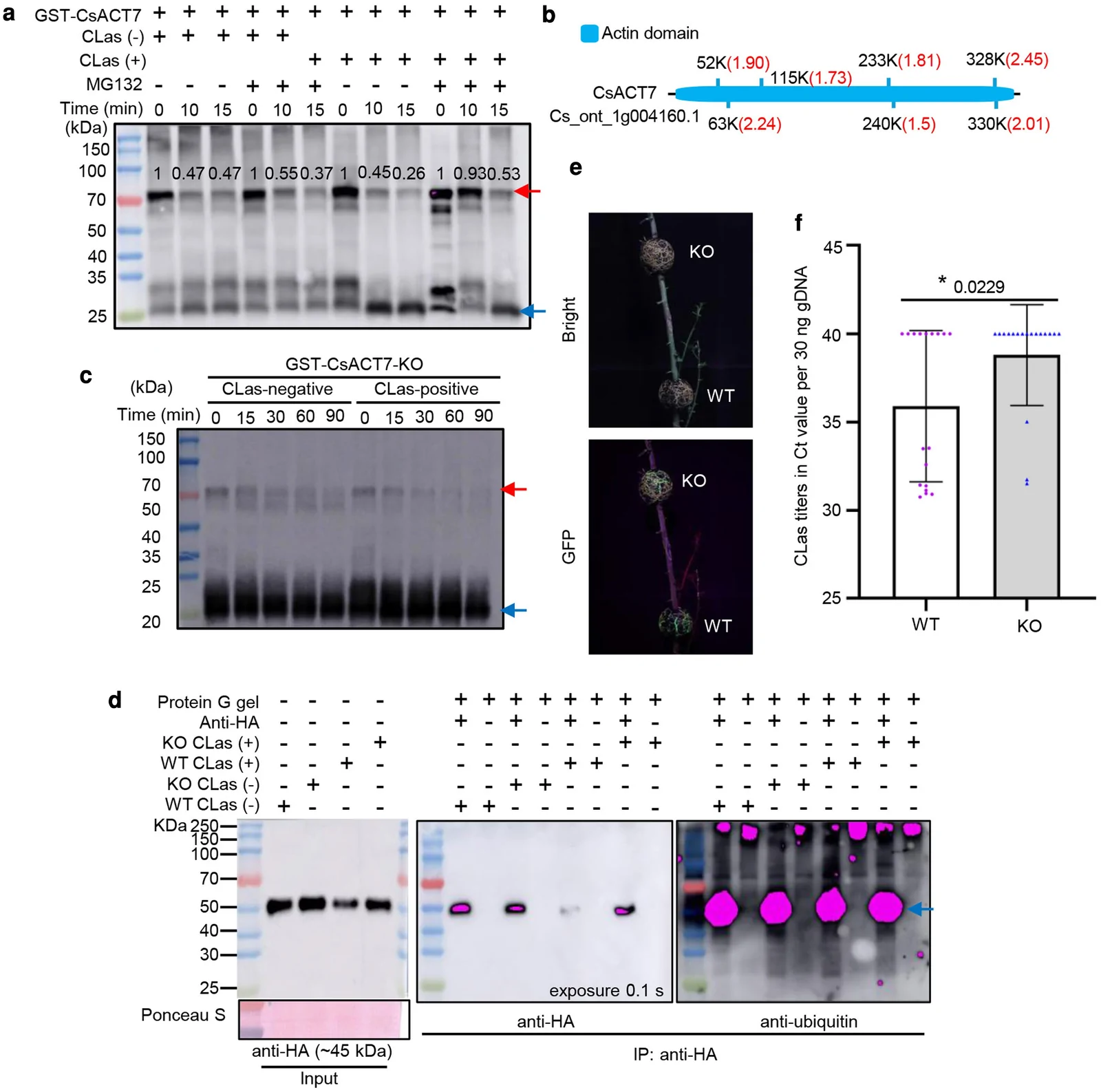
Multisite ubiquitination promotes 26S proteasome-dependent degradation of CsACT7 and contributes to CLas susceptibility. a) In vitro degradation assay showing that CLas-induced degradation of GST-CsACT7 is suppressed by MG132. Recombinant GST-CsACT7 was incubated with total protein extracts from CLas-positive or CLas-negative leaves at 25 ℃ for the indicated times in the presence or absence of 50 μM MG132, followed by immunoblot detection with an anti-GST antibody. Blue arrow, GST; red arrow, GST-CsACT7. b) Schematic diagram of the 7 ubiquitinated lysine residues identified in CsACT7 from CLas-infected leaves. Red numbers in parentheses indicate the relative increase in ubiquitination level at each site. c) In vitro degradation assay showing that the septuple lysine mutant GST-CsACT7-KO is more resistant to proteolysis than wild-type GST-CsACT7. In GST-CsACT7-KO, K52, K63, K115, K233, K240, K328, and K330 were substituted with alanine. d) Immunoprecipitation-immunoblot analysis of CsACT7 ubiquitination in transgenic *C. trifoliata* hairy roots expressing 35S::*CsACT7*-3HA (WT) or 35S::*CsACT7-KO*-3HA (KO), generated by high-pressure propagation breeding (HPPB)/Agrobacterium-mediated transformation. Total proteins extracted from WT and KO transgenic roots, with or without CLas infection, were immunoprecipitated using Protein A/G beads crosslinked with anti-HA antibody. The immunoprecipitated proteins were detected by immunoblotting with anti-HA and anti-ubiquitin antibodies, respectively. Note: The representative immunoblot images presented in this panel are derived from the full exposure time series provided in Figure S13. Specifically, the anti-HA panel displays the 0.1 s exposure, and the anti-ubiquitin panel displays the 5 s exposure from the identical blots. e) Representative image showing WT- and KO-expressing transgenic hairy roots generated simultaneously on the same CLas-positive tree by HPPB. f) CLas titer in transgenic WT and KO hairy roots. Hairy roots were sampled approximately 6 mo after HPPB. CLas titers were determined by qPCR and are shown as Ct values per 30 ng genomic DNA. Data represent mean ± SD (*n* = 18 biologically independent roots). Statistical significance was assessed by unpaired 2-tailed *t*-test. *P* < 0.05. The experiment was repeated 3 times with similar results. Lower Ct values indicate higher CLas titers.

Actin immunoprecipitation followed by anti-ubiquitin immunoblotting detected ubiquitinated actin in both CLas-negative and CLas-positive samples, with stronger signals in infected leaves (Fig. 4c), indicating enhanced actin ubiquitination (Table 3). Degradation of GST-CsACT7 in infected extracts was strongly suppressed by MG132 (Fig. 7a), consistent with 26S proteasome dependence. Mapping of CsACT7 ubiquitination sites identified 7 lysines (K52, K63, K115, K233, K240, K328, K330), all up-ubiquitinated in infected tissue (Fig. 7b). Single mutants and the K328/330A double mutant remained degradable (Figure S12, a to h), whereas a septuple mutant (CsACT7-KO) was largely resistant (Fig. 7c), indicating cooperative multisite ubiquitination.

Using the rapid HPPB/Agrobacterium hairy-root system (Zhang et al. 2025), we expressed 35S::*CsACT7*-3HA (WT) or 35S::*CsACT7*-KO-3HA (K52/63/115/233/240/328/330A) in CLas-negative and CLas-positive roots. In infected roots, CsACT7 degradation was alleviated in the KO lines relative to WT, and the enhanced ubiquitination signal was reduced but still detectable (Fig. 7d and Figure S13). qPCR revealed lower CLas titers in KO roots than in WT roots (Fig. 7, e and f).

Together, these results identify actin as a major downstream target of CLas-induced UPS remodeling and show that multisite ubiquitination-dependent CsACT7 degradation drives cytoskeletal disruption and enhances pathogen susceptibility.

## Discussion

Our study adds a posttranslational dimension to current models of HLB pathogenesis. Previous work in susceptible sweet orange showed that HLB alters photosynthesis, defense, and metabolism at the transcript and protein levels (Fan et al. 2011; Martinelli et al. 2016; Franco et al. 2020; Li et al. 2021; Gao et al. 2023). Here, we show that CLas infection also drives extensive remodeling of host protein ubiquitination and of the degradation machinery itself. CLas shifted ubiquitination strongly upward (1,676 upregulated vs. 32 downregulated Kub sites), in contrast to the psyllid vector *D. citri* (53 upregulated vs. 290 downregulated sites) (Zhang et al. 2023), highlighting host-context dependence. These data identify ubiquitin-dependent proteostasis remodeling as a major component of host response to CLas infection.

The integrated proteome–ubiquitinome analysis suggests 2 broad modes of CLas-associated ubiquitination. One features increased ubiquitination with reduced protein abundance, enriched for chloroplast function, antioxidant activity, and central carbon metabolism (Fig. 2, d and e). This pattern is consistent with ubiquitin-associated turnover contributing to loss of photosynthetic capacity, redox buffering, and metabolic homeostasis, observed with HLB symptoms (Wang et al. 2017). This view aligns with the CLas effector SDE19, which suppresses citrus immunity by 26S proteasome-dependent destabilization of Sec12 (Huang et al. 2024). The second mode includes proteins that increase in both abundance and ubiquitination and are enriched in vesicle trafficking, stress-associated metabolism, and carbohydrate metabolism (Fig. 2, b and c). This suggests non-degradative roles of ubiquitination in regulating trafficking, interactions, activity, or localization—functions well established in animals but less explored in plants (Zhou and Zeng 2017; Rehwinkel and Gack 2020). Thus, infection-associated ubiquitination likely combines degradative and regulatory components, and elevated ubiquitination alone does not imply proteasomal turnover for every substrate.

Our data indicate that CLas infection reshapes the UPS at multiple levels. Beyond widespread changes in putative substrates, increased ubiquitination of proteasome subunits and detection of E1/E2/E3 and deubiquitination-related proteins in the ubiquitinome suggest reprogramming of the degradation machinery itself (Tables 1 and 2). This remodeling coincides with enhanced proteasome-dependent bulk protein turnover (Fig. 4d), consistent with a net proteome-depleting state in infected leaves. Although the functional consequences of proteasome subunit ubiquitination remain unclear, they may affect proteasome assembly, substrate processing, localization, or stress responsiveness, providing a framework for the widespread protein loss observed in our multi-omics data.

A major consequence of this remodeling is suppression of antioxidant capacity (Figure S1). Antioxidant proteins were enriched among those with reduced abundance and increased ubiquitination, indicating posttranslational compromise of the H_2_O_2_-scavenging network (Figs. 2d and 5a; Figures S7, a to d, and S8; Dataset S16). This adds a posttranslational dimension to transcriptome studies showing antioxidant suppression in HLB-affected sweet orange (Ma et al. 2022b). Catalase emerged as 1 target, linking enhanced ubiquitination to reduced detoxification and elevated oxidative stress (Figs. 4b and 5, b to h). In *Arabidopsis*, H_2_O_2_ and SA reinforce one another during immune signaling, while prolonged ROS accumulation suppresses growth and perturbs hormone balance (Yuan et al. 2017; Cao et al. 2024). HLB-affected citrus shows elevated SA, reduced auxin, and persistent H_2_O_2_ accumulation (Pitino et al. 2017; Ma et al. 2022b; Neupane et al. 2023), suggesting that antioxidant depletion drives a maladaptive redox state that amplifies defense-associated injury. Together with global proteasome-dependent protein loss, these results indicate a broad degradative shift in host proteostasis.

A second major advance is the identification of actin as a downstream target of CLas-induced UPS remodeling. Cytoskeletal disruption is common in plant–pathogen interactions, but our data support a specific mechanism in which host actin is destabilized by multisite ubiquitination and 26S proteasome-dependent degradation (Figs. 6 and 7). CsACT7 is unlikely to be the only isoform involved: the 7 ubiquitinated lysines required for CsACT7 destabilization are highly conserved among other citrus actins (Figure S14), corresponding sites are ubiquitinated in CsACT3/4/8 (Dataset S15), and GST-CsACT4 and GST-CsACT8 are also degraded by infected-leaf extracts in vitro (Fig. 6, j and l). Because all *CsACT* genes are expressed in leaves (Figure S9c), CLas-induced actin turnover and microfilament disruption likely involve multiple isoforms, with CsACT7 providing the clearest mechanistic example.

Actin dynamics are central to vesicle trafficking, organelle positioning, cell integrity, and immune signaling, and many pathogens perturb host actin to manipulate these processes (Kang et al. 2014; Li and Day 2019). Unlike bacterial pathogens whose type III effectors directly target actin (Kang et al. 2014; Hiles et al. 2024), CLas likely achieves similar outcomes indirectly via host UPS-dependent actin turnover, a strategy also reported in *D. citri* midguts during CLas colonization (Ghanim et al. 2016). Because no CLas effector with E3 ligase activity has been identified (Dataset S17), a plausible model is that CLas rewires host signaling to activate endogenous actin-directed ubiquitination. Consistent with this, SDE5 potentiates the citrus U-box ligase PUB21 toward MYC2 to promote ubiquitin-dependent immune suppression (Zhao et al. 2025).

The actin phenotype may intersect with redox and hormone disturbances in HLB. In plants, actin depolymerization amplifies flg22-triggered ROS and SA production, and SA and H_2_O_2_ reciprocally induce cytoskeletal remodeling (Matoušková et al. 2014; Li et al. 2017; Leontovyčová et al. 2019). In mammals, actin disruption can prime RIG-I-like receptor signaling via PPP1R12C relocalization and PP1-dependent pathways (Acharya et al. 2022). In HLB-affected citrus, CLas infection is associated with SA accumulation (Neupane et al. 2023), H_2_O_2_ overproduction (Pitino et al. 2017; Ma et al. 2022b), and irreversible actin degradation (Fig. 6m). Together, these observations suggest a self-reinforcing loop among SA, ROS, and cytoskeletal destabilization that may sustain immune hyperactivation and growth suppression. However, our data do not resolve causality; defining the temporal and spatial hierarchy of SA–H_2_O_2_–cytoskeleton crosstalk remains a key goal.

Overall, our findings support a model in which CLas infection drives broad, maladaptive reprogramming of citrus proteostasis. Ubiquitination contributes to depletion of chloroplast, antioxidant, and metabolic proteins while modulating a distinct set of stress-responsive and trafficking-related proteins. Remodeling of the proteasome and other ubiquitin-system components, together with MG132-sensitive bulk protein loss, indicates functional reconfiguration of the degradation machinery. Two major outputs are suppression of antioxidant capacity with pathological H_2_O_2_ accumulation and actin cytoskeletal disruption through multisite ubiquitination-dependent degradation, with CsACT7 the strongest validated example. These processes likely integrate with previously described transcriptional, metabolic, and immune perturbations to reinforce phloem dysfunction and symptom progression.

Several questions remain. The upstream signals linking CLas perception to global UPS remodeling are unknown, as are the E3 ligases and DUBs acting on key substrates such as catalase, APX isoforms, and actin. It will also be important to define ubiquitin-chain topologies and to distinguish proteasomal from autophagic turnover in infected tissues. Finally, because stabilization of CsACT7 reduced CLas populations in hairy roots (Fig. 7f), manipulating host proteostasis may offer a conceptual route for HLB mitigation. More broadly, this study establishes ubiquitination-dependent proteome remodeling as a central feature of the citrus response to CLas and provides a mechanistic framework linking posttranslational regulation to oxidative stress, bulk protein depletion, cytoskeletal disruption, and disease susceptibility.

## Materials and methods

### Genomic and proteomic resources

All citrus sequences were obtained from the Citrus Pan-genome to Breeding Database (CPBD, http://citrus.hzau.edu.cn). The *Citrus sinensis* v3.0 reference genome served as the primary source for genomic sequences and annotations, unless otherwise specified. The CPBD provided curated protein-coding sequences and functional annotations for homology analyses. Protein sequence alignments were performed using BLASTP (*E*-value cutoff: 1e^−5^) to verify orthologous relationships and domain conservation.

### Plant material and DNA analysis

Seven-year-old Newhall navel orange (*C. sinensis*) grafted onto Carrizo citrange (*C. sinensis* × *Citrus trifoliata*) rootstocks were cultivated in an orchard in Ganzhou, China (25°46′23″N, 114°53′9″E). Fully expanded leaves (approximately 60 d post-bud initiation), stems, and roots of comparable developmental stages were collected from HLB-infected and healthy plants. HLB infection status was confirmed by quantitative PCR (qPCR) targeting the CLas *β-subunit of the ribonucleotide reductase* (*RNR*) gene using primer pair RNRf/RNRr (Table S1) (Zheng et al. 2016). Genomic DNA was extracted from 0.2 g tissue using the E.Z.N.A. HP Plant DNA Kit (Omega Bio-tek, D2485-02).

### Protein extraction

Leaf tissue (2 g) was flash-frozen in liquid nitrogen, ground to a powder, and homogenized in 4 volumes of ice-cold trichloroacetic acid (TCA)–acetone for 4 h at −20 °C. Precipitated proteins were pelleted (5,500 × g, 10 min, 4 °C), washed with acetone, and resuspended in SDS lysis buffer (50 mM Tris-HCl pH 7.5, 2% SDS, 10 mM DTT, 1 × protease inhibitor cocktail, 50 μM PR-619). The lysate was sonicated on ice (5 cycles of 30 s ON/30 s OFF) and extracted with Tris-saturated phenol (pH 8.0). Proteins were precipitated with 0.1 M ammonium acetate/methanol, washed sequentially with methanol and acetone, and dissolved in 8 M urea. Protein concentrations were quantified using a BCA assay (Beyotime Biotechnology, P0009).

### Trypsin digestion

For each sample, 100 μg of protein was precipitated with 20% TCA, washed with pre-cooled acetone, and resuspended in 200 mM tetraethylammonium bromide and sonicated. Proteins were digested overnight with trypsin (1:50 wt/wt; V5111, Promega) at 37 °C. Tryptic peptides were reduced with 5 mM DTT (56 °C, 30 min) and alkylated with 11 mM iodoacetamide (room temperature, 15 min, dark).

### Ubiquitinated peptide enrichment

Digested peptides were dissolved in NETN buffer (100 mM NaCl, 1 mM EDTA, 50 mM Tris-HCl pH 8.0, 0.5% NP-40) and incubated overnight at 4 °C with anti-K-ε-GG antibody-conjugated resin (PTM-1104, PTM Biolabs). After washing, bound peptides were eluted with 0.1% trifluoroacetic acid, desalted using ZipTip C_18_ (Millipore, 41121500), and vacuum-dried.

### Liquid chromatography-tandem mass spectrometry

Peptides were dissolved in solvent A (0.1% formic acid, 2% acetonitrile) and separated using a reverse-phase analytical column (75 μm inner diameter, 15 cm length) coupled to the NanoElute ultra-high performance liquid chromatography system. A gradient of solvent B (0.1% formic acid, 100% acetonitrile) was applied as follows: 6% to 22% B over 44 min, 22% to 30% B over 10 min, 30% to 80% B in 4 min, followed by a 4-min hold at 80% B, with a constant flow rate of 450 nL/min. Ionization was achieved via a capillary ion source (1.7 kV). Precursor ions and collision-induced dissociation fragments were analyzed using a time-of-flight mass spectrometer operated in parallel accumulation serial fragmentation (PASEF) mode. MS/MS scans (100 to 1,700 m/z) were acquired with 10 PASEF events per cycle, employing a 30-s dynamic exclusion window to minimize redundant precursor selection.

### Database search

Raw MS/MS spectra were processed in MaxQuant (v1.6.15.0) and searched against the *C. sinensis* protein database (*Citrus sinensis* 2711 CPBD SWO.v3.0 20211224 seqkit.fasta; 42,321 entries), supplemented with a reverse decoy database for false discovery rate (FDR) calculation and a common contaminant database to filter out nonspecific hits. Trypsin/P was designated as the protease (maximum 4 missed cleavages), with peptide length ≥ 7 residues and ≤5 modifications per peptide. Mass tolerances were set to 20 ppm for both precursor and fragment ions. Fixed modifications included carbamidomethylation of cysteine, while variable modifications comprised methionine oxidation, N-terminal acetylation, and lysine ubiquitination. Protein and peptide-spectrum match (PSM) identifications were filtered at a 1% FDR threshold, with proteins requiring ≥1 unique peptide for confident assignment.

### Bioinformatics analysis

GO annotations were retrieved from the UniProt-GOA database (http://www.ebi.ac.uk/GOA/), and unannotated sequences were functionally classified using InterProScan based on sequence homology. DEPs and ubiquitinated sites were identified with a fold change (FC) > 1.5 and *P* < 0.05, confirmed across at least 2 of 3 biological replicates. Proteins were classified by GO annotation into 3 categories: biological process, cellular compartment, and molecular function. Fisher's exact test was conducted for each category to assess the enrichment of differentially expressed/ubiquitinated proteins against all identified proteins, with a corrected *P*-value < 0.05 considered significant. Subcellular localization was predicted using WoLF PSORT, and Motif-X software (http://meme-suite.org/tools/momo) was used for identifying ubiquitination motifs.

### Homolog identification and phylogenetic tree construction

*C. sinensis* homologs of *Arabidopsis* proteins were identified via BLAST against the *C. sinensis* V3.0 database (http://citrus.hzau.edu.cn/). Proteins lacking conserved domains, as predicted by the Conserved Domain Database, were excluded from further analysis. Phylogenetic trees were constructed using amino acid sequences. The maximum likelihood method was employed for tree construction, with the Whelan and Goldman (WAG) model applied to account for amino acid substitution rates. Tree topology reliability was assessed using the bootstrap method. All other parameters were set to default values in MEGA11 software.

### Plasmid construction and protein purification

The coding sequence of *CsACT7* was amplified from Hamlin leaf cDNA and initially cloned into pDONR207 using Gateway BP Clonase II Enzyme mix (Thermo Fisher Scientific, 11789100). The sequence was subsequently transferred into pDEST15 via Gateway LR Clonase II Enzyme mix (Thermo Fisher Scientific, 11791100) to generate a GST fusion construct. Site-directed mutagenesis was employed to create lysine-to-alanine variants of GST-CsACT7. Recombinant proteins were expressed in *Escherichia coli* BL21 using 0.5 mM IPTG for 4 hours at 28 °C. Proteins were purified using BeyoGold GST-tag Purification Resin (Beyotime Biotechnology, P2253) according to the manufacturer's instructions.

### Genetic transformation

To visualize the actin cytoskeleton, transgenic roots expressing *EYFP-Lifeact* were generated using the hairy root genetic transformation method (Ma et al. 2022a). The *UBQ10pro::EYFP-Lifeact* construct was introduced into *Agrobacterium rhizogenes* K599 via electroporation. To efficiently obtain both CLas-positive and CLas-negative transgenic roots expressing EYFP-Lifeact, both HLB-infected and healthy *C. trifoliata* stems were utilized for transformation. This approach avoided the time-consuming and low-efficiency methods of CLas transmission via psyllid inoculation or grafting. Six months posttransformation, the CLas status of individual transgenic roots was determined by qPCR.

To generate transgenic hairy roots expressing 35S::*CsACT7*-3HA (WT) or 35S::*CsACT7*-KO-3HA (KO), an HPPB-mediated *A. rhizogenes* K599 transformation system was used as described previously (Zhang et al. 2025). Because CLas is restricted to the phloem, transformation was performed on the same CLas-positive *C. trifoliata* plant to maintain the consistency of CLas transmission to WT and KO roots. The coding sequences of CsACT7 and CsACT7-KO were cloned into pCAMBIA1380 vector using In-Fusion HD Cloning kits (Takara, 639650). Regenerated roots were preliminarily screened by GFP fluorescence using a portable excitation light source, and each GFP-positive root was collected individually for subsequent analyses.

### In vitro degradation assays

Mortars and pestles were prechilled at −20 °C. Leaf tissue (0.1 g) from healthy and HLB-infected Newhall was homogenized in cold extraction buffer (50 mM Tris-HCl pH 7.5, 150 mM NaCl, 5 mM EDTA, 0.1% Triton X-100, 0.2% NP-40, 1 × protease inhibitor cocktail) and clarified by centrifugation (12,000 × *g*, 10 min, 4 °C). Protein concentration was quantified using the Bradford Dye method (Bio-Rad, 5000205) and adjusted to equal concentrations. Purified GST-CsACT7 variants (9 μL) were incubated with 9 μL of total protein extract and 2 μL of 10 × protease inhibitor cocktail at 25 °C for the indicated time. Reactions were terminated by adding 5 × SDS loading buffer, followed by heating at 85 °C for 10 min in a metal block. Immunoblots were performed using anti-GST antibody (TransGen Biotech, HT601-01). Due to the uneven distribution of CLas within a citrus tree, the bacterial titer of CLas in each leaf was individually confirmed using qPCR.

### Reverse transcription quantitative PCR

Total RNA was isolated using TRIzol (Invitrogen, 15596018CN). cDNA was synthesized from 500 ng of total RNA using PrimeScript RT Master Mix (Takara, RR036A) and diluted 10 times for use. Reverse transcription quantitative PCR (RT-qPCR) was performed using TB Green Premix Ex Taq II (Takara, RR092A). *CsGAPDH* served as the endogenous control. Relative expressions were calculated using the 2^−△△CT^ method. Primers used for RT-qPCR are listed in Table S1. Data represent 3 biological replicates.

### Antibody crosslinking to Protein A/G agarose

To reduce contamination by IgG heavy and light chains during immunoprecipitation, anti-actin, anti-CAT2, and anti-RPT2B antibodies were covalently crosslinked to Protein A/G agarose using disuccinimidyl suberate (DSS). Protein A/G agarose slurry (50%; 60 μL, equivalent to 30 μL packed beads) was washed twice with PBS and twice with 0.1 M sodium borate buffer (pH 8.3). Anti-actin (PTM BIO, PTM-6702), anti-CAT2 (PhytoAB, PHY3044A), anti-RPT2B antibody (PhytoAB, PHY3246A), or anti-HA (Proteintech, 51064-2-AP) (4 μg) was incubated with the beads in 500 μL borate buffer at 4 °C for 2 h. After washing, antibody-bound beads were incubated with 1 mM freshly prepared DSS in 0.1 M sodium borate buffer (pH 8.3) for 1 h at room temperature with gentle rotation. The reaction was quenched with 0.5 M Tris-HCl (pH 8.0) for 20 min, and beads were washed 5 times with PBS. Residual non-crosslinked antibody was removed by brief washing with 0.1 M glycine (pH 2.5), followed by immediate neutralization with 1 M Tris-HCl (pH 9.0). Beads were resuspended in PBS and stored at 4 °C until use.

### In vivo ubiquitination assays

In vivo ubiquitination assays were performed under ubiquitin-preserving immunoprecipitation conditions. Fresh leaves from CLas-positive and CLas-negative Newhall trees at comparable developmental stages were collected and processed on ice. Because of the total protein degradation induced by CLas infection, 0.7 g CLas-positive and 1.0 g CLas-negative leaves were extracted in 5 mL of prechilled IP buffer containing 50 mM Tris-HCl (pH 7.5), 150 mM NaCl, 5 mM EDTA, 0.5% Triton X-100, 0.5% NP-40, 1 mM DTT, 0.5% PVPP, 10 mM *N*-ethylmaleimide, and 1 × protease inhibitor cocktail. Extracts were clarified by centrifugation, and protein concentration was determined using Bradford reagent (Bio-Rad, 5000201).

For immunoprecipitation, anti-Actin, anti-CAT2, anti-RPT2B, and anti-HA antibodies were crosslinked to Protein A/G agarose as described above. Immunoprecipitates were washed, eluted in 2 × SDS loading buffer with 50 mM DTT, separated by SDS-PAGE, and analyzed by immunoblotting. Ubiquitinated proteins were detected with anti-ubiquitin (PTM BIO, PTM-1106RM) antibody. Input samples were analyzed in parallel.

### H_2_O_2_ extraction and quantification

H_2_O_2_ was quantified using a potassium iodide-based colorimetric assay as described previously (Ma et al. 2022b). Fresh sweet orange leaves (0.2 g) were homogenized in 0.2 mL ice-cold 0.1% (wt/vol) TCA and centrifuged at 12,000 × *g* for 15 min at 4 °C. The supernatant (20 μL) was mixed with 113 μL 1.0 M potassium phosphate buffer (pH 7.0) and 67 μL 1.0 M KI in a 96-well plate. After incubation for 5 min at room temperature, absorbance was measured at 390 nm. A standard curve was generated using freshly prepared diluted H_2_O_2_ in 0.1% TCA and processed in parallel. H_2_O_2_ content was calculated from the standard curve and expressed as μmol g^−1^ fresh weight. Data represent 3 biological replicates.

## Supplementary Material

kiag612_Supplementary_Data

## Data Availability

All data can be found within the manuscript and supporting materials, and the raw MS data are available via ProteomeXchange with the dataset identifier PXD062872.
