# Supplementary material for: Ubiquitination-driven proteostasis remodeling underlies oxidative stress and actin degradation in citrus huanglongbing
Source: Plant Physiol. 2026 Aug 25;202(1):kiag612. doi: 10.1093/plphys/kiag612 (PMC13530299; doi:10.1093/plphys/kiag612)
Supplement: Supplementary file 1 [file kiag612_supplementary_data.zip › Supplementary-Manuscript-revised-NOhighlight-TX-4th.pdf]

Supporting Information for

Ubiquitination-driven proteostasis remodeling underlies oxidative stress and actin degradation in citrus Huanglongbing

Min Li[a, b, 1, *], Hongkun Qin[a, 1], Yundan Hu[a, 1], Bingqian Qin[a], Zirui Fan[a], Mingzhuo Li[a], Yaping Mao[a], Fajin Liao[a], Zhigang Ouyang[a, b], Huan Chen[c], Shuo Duan[a, b, *]

[a]National Navel Orange Engineering Research Center, College of Life Sciences, Gannan Normal University, Ganzhou 341000, China.

[b]Jiangxi Provincial Key Laboratory of Pest and Disease Control of Featured Horticultural Plants, Gannan Normal University, Ganzhou 341000, China.

[c]School of Agriculture and Biology, Shanghai Jiao Tong University, Shanghai, 200240, China.

[1]These authors contributed equally: Min Li, Hongkun Qin and Yundan Hu.

*To whom correspondence may be addressed.

Email: minli727@gnnu.edu.cn

duanshuo@gnnu.edu.cn

The author responsible for distribution of materials integral to the findings presented in this article in accordance with the policy described in the Instructions for Authors (https://academic.oup.com/plphys/pages/General-Instructions) is (Shuo Duan).

Running title: Proteostasis Remodeling in Citrus HLB

Keywords: Huanglongbing, citrus greening disease, *Candidatus* Liberibacter asiaticus, ubiquitination, ubiquitin-proteasome system, antioxidant, actin

This PDF file includes:

Figures S1 to S14

Legends for Datasets S1 to S17

Tables S1

Other supporting materials for this manuscript include the following:

Datasets S1 to S17

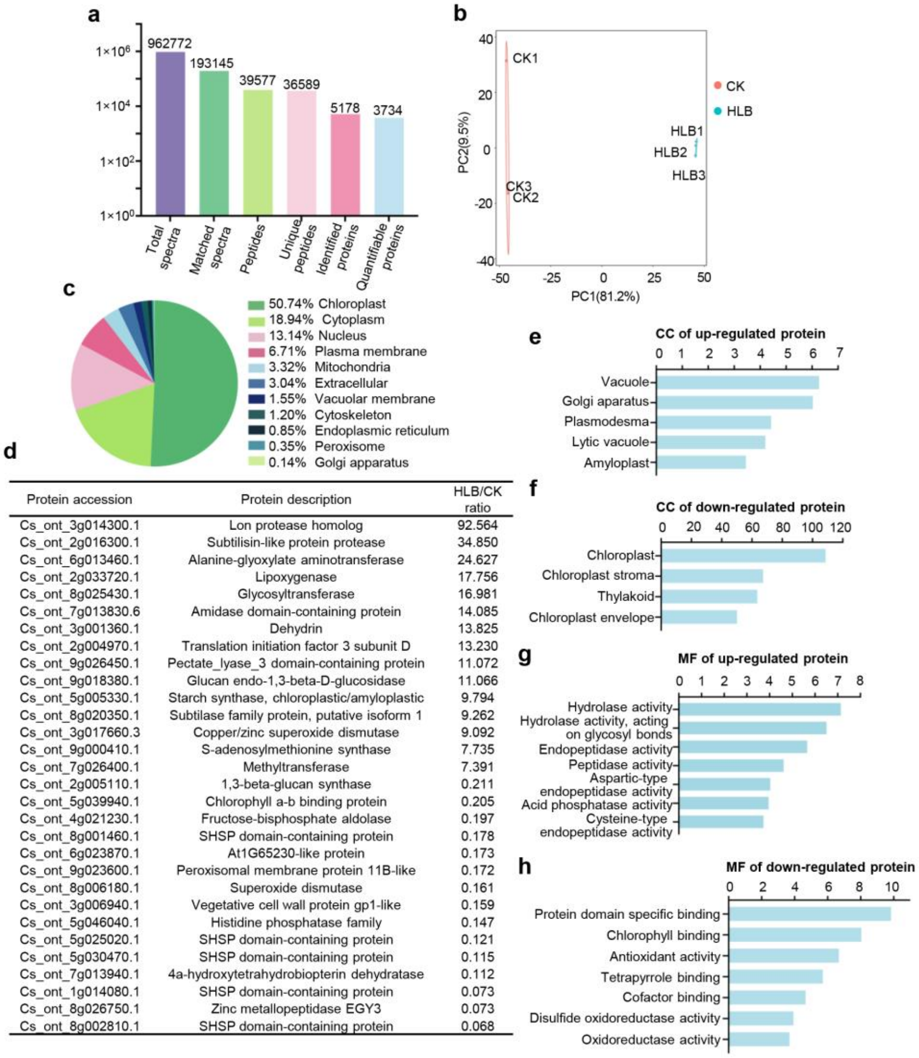

Protein accession	Protein description	HLB/CK ratio
Cs_ont_3g014300.1	Lon protease homolog	92.564
Cs_ont_2g016300.1	Subtilisin-like protein protease	34.850
Cs_ont_6g013460.1	Alanine-glyoxylate aminotransferase	24.627
Cs_ont_2g033720.1	Lipoxygenase	17.756
Cs_ont_8g025430.1	Glycosyltransferase	16.981
Cs_ont_7g013830.6	Amidase domain-containing protein	14.085
Cs_ont_3g001360.1	Dehydrin	13.825
Cs_ont_2g004970.1	Translation initiation factor 3 subunit D	13.230
Cs_ont_9g026450.1	Pectate_lyase_3 domain-containing protein	11.072
Cs_ont_9g018380.1	Glucan endo-1,3-beta-D-glucosidase	11.066
Cs_ont_5g005330.1	Starch synthase, chloroplastic/amyloplastic	9.794
Cs_ont_8g020350.1	Subtilase family protein, putative isoform 1	9.262
Cs_ont_3g017660.3	Copper/zinc superoxide dismutase	9.092
Cs_ont_9g000410.1	S-adenosylmethionine synthase	7.735
Cs_ont_7g026400.1	Methyltransferase	7.391
Cs_ont_2g005110.1	1,3-beta-glucan synthase	0.211
Cs_ont_5g039940.1	Chlorophyll a-b binding protein	0.205
Cs_ont_4g021230.1	Fructose-bisphosphate aldolase	0.197
Cs_ont_8g001460.1	SHSP domain-containing protein	0.178
Cs_ont_6g023870.1	At1G65230-like protein	0.173
Cs_ont_9g023600.1	Peroxisomal membrane protein 11B-like	0.172
Cs_ont_8g006180.1	Superoxide dismutase	0.161
Cs_ont_3g006940.1	Vegetative cell wall protein gp1-like	0.159
Cs_ont_5g046040.1	Histidine phosphatase family	0.147
Cs_ont_5g025020.1	SHSP domain-containing protein	0.121
Cs_ont_5g030470.1	SHSP domain-containing protein	0.115
Cs_ont_7g013940.1	4a-hydroxytetrahydrobiopterin dehydratase	0.112
Cs_ont_1g014080.1	SHSP domain-containing protein	0.073
Cs_ont_8g026750.1	Zinc metallopeptidase EGY3	0.073
Cs_ont_8g002810.1	SHSP domain-containing protein	0.068

Figure S1. Global proteomic features and functional classification of DEPs in CLas-infected Newhall leaves. (a) Overview of protein identification in the proteome. **(b)** Principal component analysis (PCA) of proteome profiles demonstrating the separation between CK and HLB leaves. **(c)** Predicted subcellular localization of DEPs. **(d)** Top 15 up and down regulated proteins in Newhall leaves under CLas infection. **(e-h)** GO term enrichment analysis of up-regulated proteins in cellular component (CC, **e**) and molecular function (MF, **g**) categories, as well as of down-regulated proteins in CC **(f)** and MF **(h)** categories. Enrichment analysis was determined against a background of all predicted proteins in *C. sinensis*. Significance was calculated using Fisher's exact test, and terms with $P < 0.05$ were considered significantly enriched. The y-axis indicates GO terms, and the x-axis shows –log10(P value).

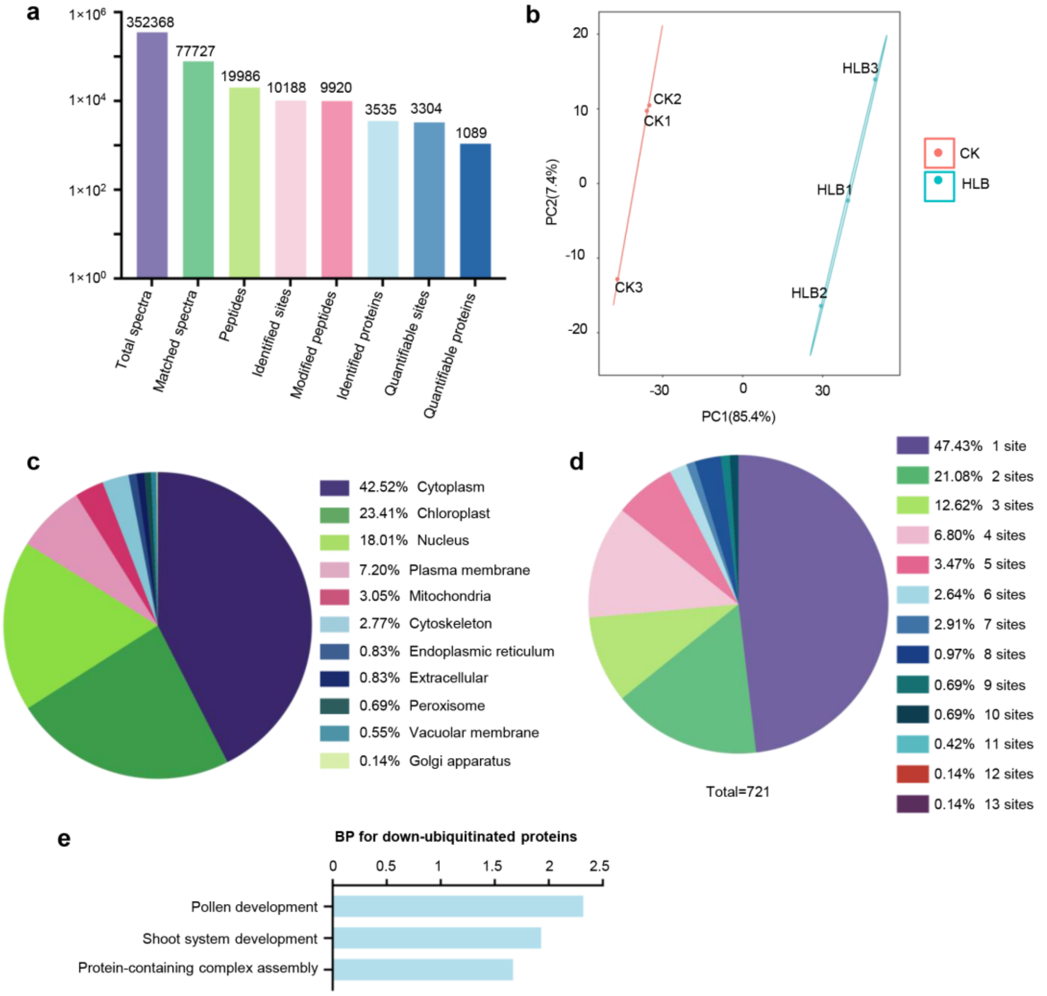

Figure S2. Ubiquitinome dynamics in CLas-infected citrus leaves. (a) Overview of protein identification in the ubiquitinome. **(b)** PCA reveals significant ubiquitination modification changes induced by CLas infection. **(c)** Distribution of DUPs categorized by their localization. **(d)** Classification of ubiquitinated proteins based on the number of modified sites. **(e)** Enrichment analysis of biological processes for DUPs showing reduced ubiquitination. The analysis was conducted against a background of all predicted proteins in *C. sinensis*. Fisher's exact test was utilized to calculate the significance P-values, with $P < 0.05$ considered statistically significant. The vertical axis represents functional categories, while the horizontal axis displays the -log10(P values).

a

Protein accession	Number of sites	Protein description
Cs_ont_8g005690.1	13	AAA domain-containing protein
Cs_ont_7g003890.1	12	Hsp70
Cs_ont_5g041680.1	11	Hsp70-Hsp90 organizing protein
Cs_ont_6g006770.1	11	UGPase
Cs_ont_6g007450.1	11	Fructose-bisphosphate aldolase
Cs_ont_5g032800.3	10	Glutathione S-transferase Tau2
Cs_ont_8g000140.2	10	E1 ubiquitin-activating enzyme
Cs_ont_8g009770.1	10	Ubiquitin carboxyl-terminal hydrolase
Cs_ont_8g013640.1	10	Diadenosine tetraphosphate synthetase
Cs_ont_9g028060.1	10	Phosphoglycerate kinase
Cs_ont_3g005880.1	9	CRAL-TRIO domain-containing protein
Cs_ont_5g014520.1	9	Methionine synthase
Cs_ont_6g007340.1	9	Phosphopyruvate hydratase
Cs_ont_8g005810.1	9	Ubiquitin
Cs_ont_9g027670.6	9	Type G domain-containing protein
Cs_ont_3g001020.1	8	Catalase
Cs_ont_4g021230.1	8	Fructose-bisphosphate aldolase
Cs_ont_5g022120.1	8	Peptidase_S9 domain-containing protein
Cs_ont_5g044290.1	8	Glyceraldehyde-3-phosphate dehydrogenase
Cs_ont_6g017180.1	8	NAD(P)-domain-containing protein
Cs_ont_9g014710.1	8	Adenosylhomocysteinase
Cs_ont_1g000790.1	7	Inositol-3-phosphate synthase
Cs_ont_1g004160.1	7	Actin
Cs_ont_1g011050.2	7	AAA domain-containing protein
Cs_ont_1g027710.1	7	Linker histone H1/H5
Cs_ont_1g028860.1	7	Phospholipase D
Cs_ont_2g035600.1	7	YTH domain-containing protein
Cs_ont_3g024380.3	7	Isocitrate dehydrogenase (NADP)
Cs_ont_4g000650.1	7	Plasma membrane ATPase
Cs_ont_4g009790.1	7	Ribosomal_L28e domain-containing protein
Cs_ont_4g022570.5	7	Sucrose synthase
Cs_ont_5g007820.1	7	Sucrose synthase
Cs_ont_5g045690.1	7	Glutaredoxin domain-containing protein
Cs_ont_5g047720.1	7	Glutathione peroxidase
Cs_ont_6g010040.1	7	Ubiquitin carboxyl-terminal hydrolase
Cs_ont_6g016560.2	7	Delta-1-pyrroline-5-carboxylate synthase
Cs_ont_7g001710.1	7	Clathrin heavy chain
Cs_ont_7g005390.2	7	Elongation factor 1-alpha
Cs_ont_7g015940.2	7	Peptidylprolyl isomerase
Cs_ont_9g002090.1	7	Bet_v_1 domain-containing protein
Cs_ont_9g008220.1	7	Malate dehydrogenase
Cs_ont_9g025000.1	7	PKS_ER domain-containing protein

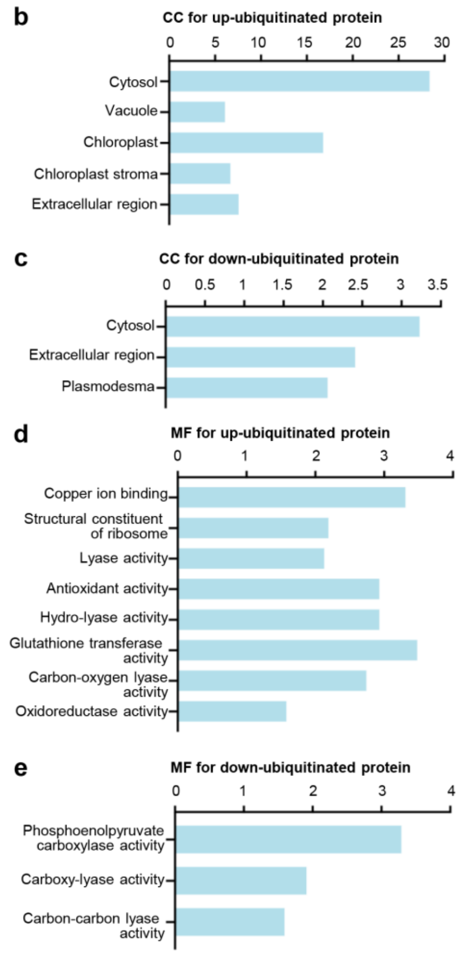

Figure S3. Functional analysis of DUPs in CLas-infected citrus leaves. (a) List of proteins with 7 or more ubiquitination sites in Newhall leaves under CLas infection. **(b-e)** GO term enrichment of proteins with up-regulated ubiquitination modification in CC **(b)** and MF **(d)**, as well as for proteins with down-regulated ubiquitination modification in CC **(c)** and MF **(e)**, was determined against a background of all predicted proteins in *C. sinensis*. Fisher's exact test was employed to calculate the significance P-values, with $P < 0.05$ considered statistically significant. The vertical axis represents GO categories, while the horizontal axis displays the -log10(P values).

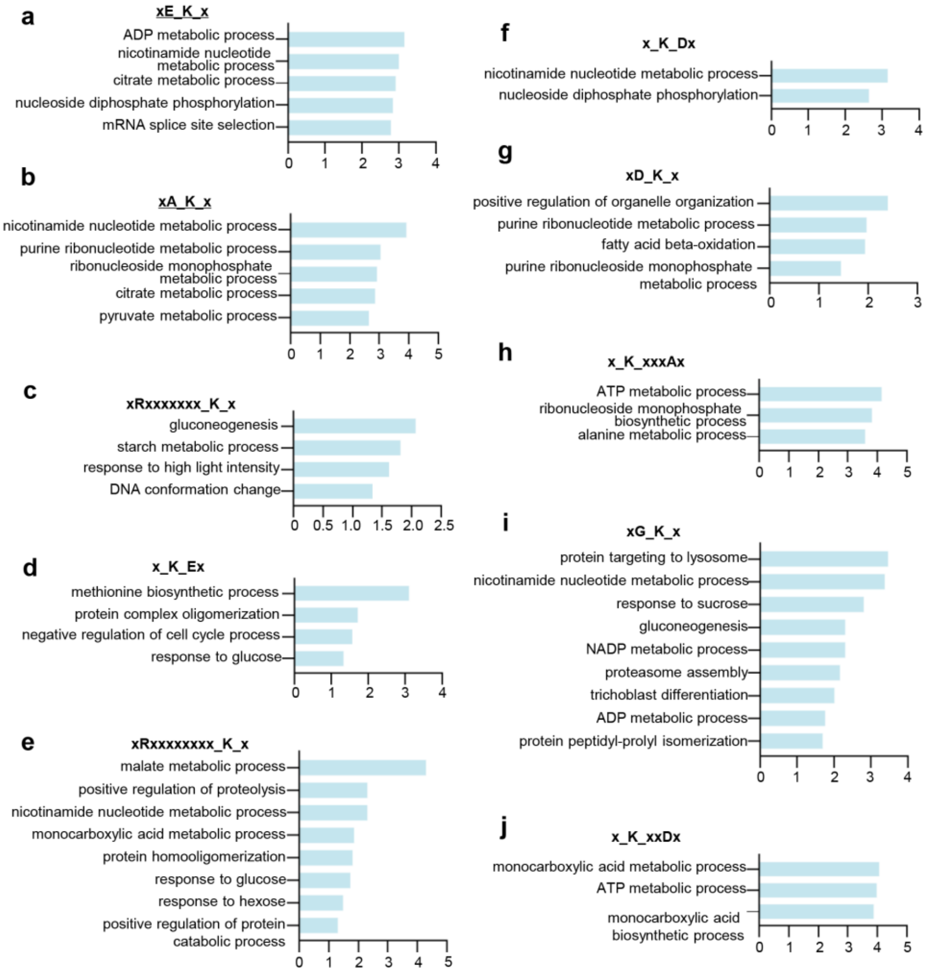

Figure S4. GO enrichment of DUPs containing enriched ubiquitination motif. (a to **j)** DUPs containing the motif identified in Fig. 3C were subjected to GO analysis in biological processes. Analyses were conducted against the proteins identified in the proteome. Fisher's exact test was utilized to compute P values, with $P < 0.05$ deemed statistically significant. The vertical axis indicates the biological processes, while the horizontal axis represents the -log10(P values).

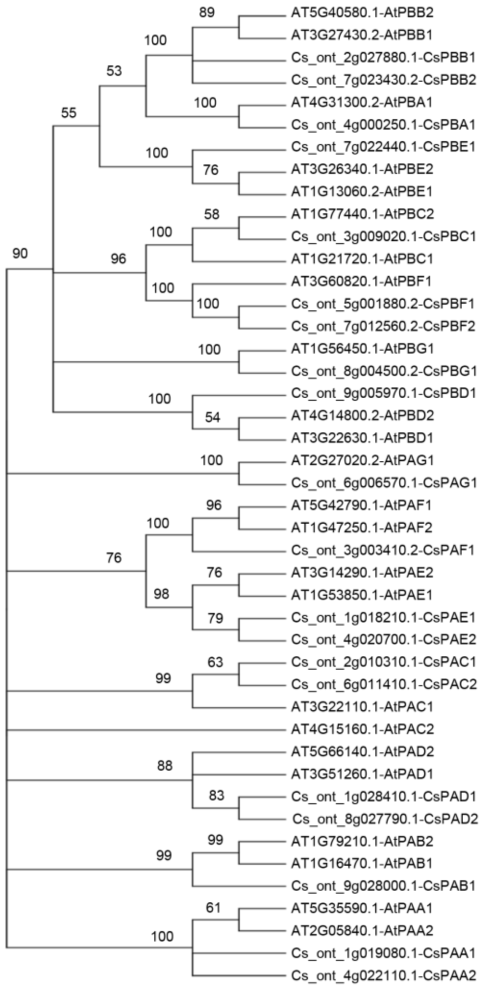

Figure S5. Phylogenetic analysis of the 20S core protease (CP) subunits of the 26S proteasome in *A. thaliana* and *C. sinensis*. Homologs in *C. sinensis* were identified by BLASTP analysis against the Citrus sinensis v3.0 protein database using the 20S CP subunits from *A. thaliana* as queries. Homologs were confirmed by conserved motif analysis via NCBI Conserved Domain Search. The phylogenetic tree was constructed using the ML method with the WAG model in MEGA11. *CsCPs* were named based on their sequence similarity to *A. thaliana* proteins and chromosomal location. All other parameters used default MEGA11 settings.

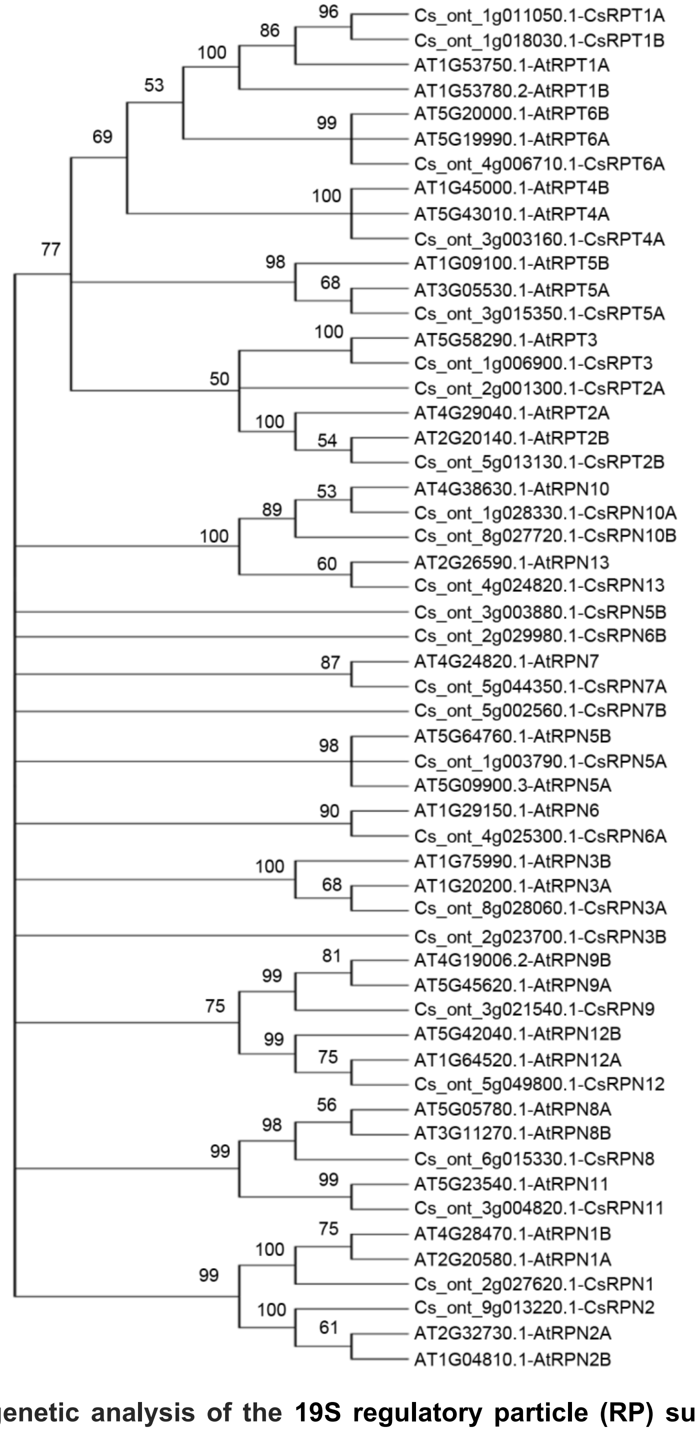

Figure S6. Phylogenetic analysis of the 19S regulatory particle (RP) subunits of the 26S proteasome in *A. thaliana* and *C. sinensis*. Identification, confirmation, tree construction, and naming followed the same procedures as in Figure S5. *CsRPs* were named based on amino acid sequence similarity to *A. thaliana* and chromosomal location.

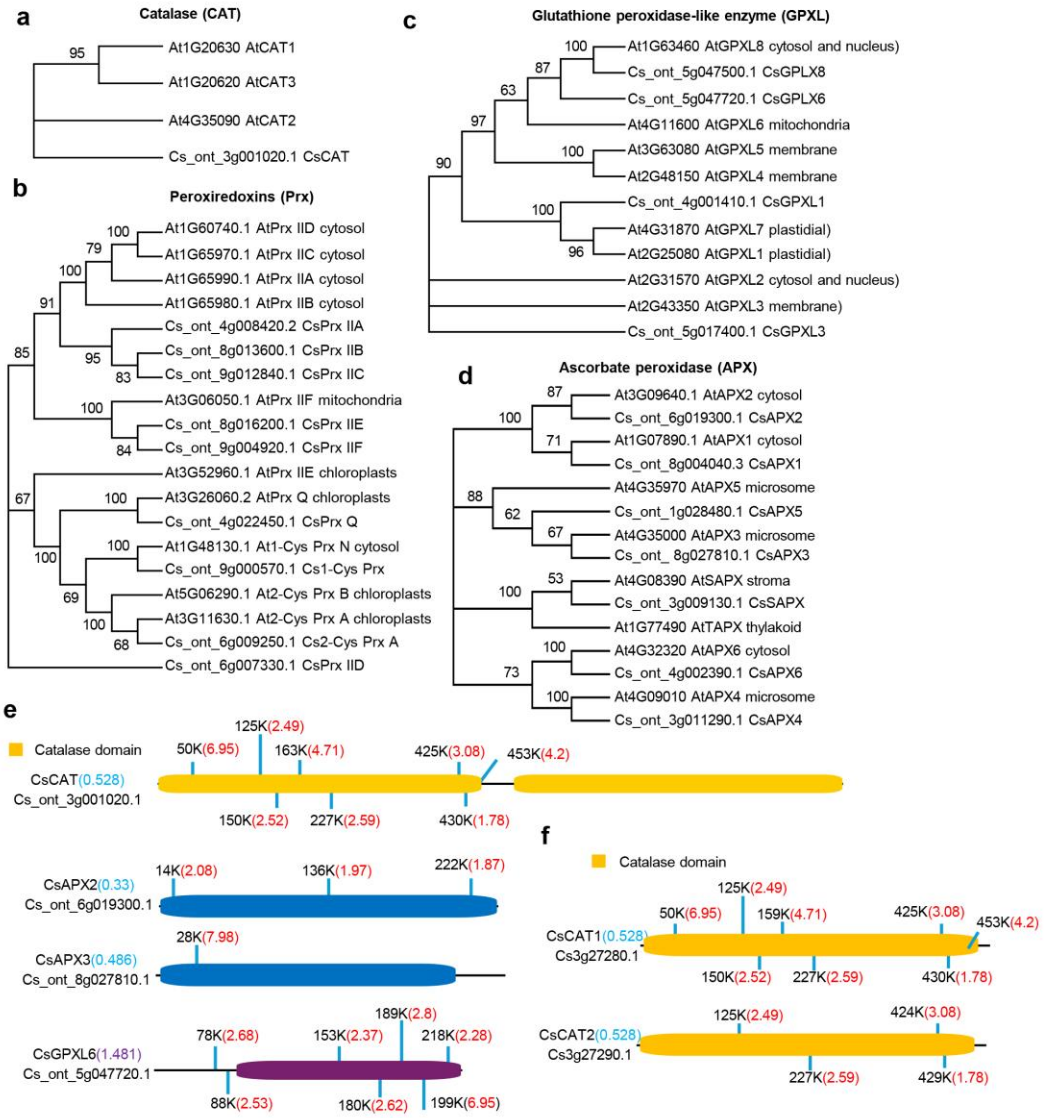

Figure S7. Phylogenetic analysis of H_2O_2-scavenging enzymes. (a to d) Phylogenetic trees of Catalase (CAT, **a**), peroxiredoxin (Prx, **b**), glutathione peroxidase-like enzyme (GPXL, **c**), ascorbate peroxidase (APX, **d**) from *A. thaliana* and *C. sinensis*. Trees were constructed using the ML method based on amino acid sequences. The statistical analysis employed the bootstrap method to assess the reliability of the tree topology. The substitution model utilized was the WAG model, which accounts for amino acid replacement rates. All other parameters were set according to the default settings of MEGA11 software. **(e)** Ubiquitination status of CsCAT, CsAPX2, CsAPX3, CsGPXL6 annotated in C. sinensis v3.0 database. Blue numbers: decreased protein abundance; purple number: increased protein abundance; red: elevated ubiquitination. **(f)** Schematic summary of ubiquitination status of CsCAT1 and CsCAT2 annotated in the C. sinensis v2.0 database.

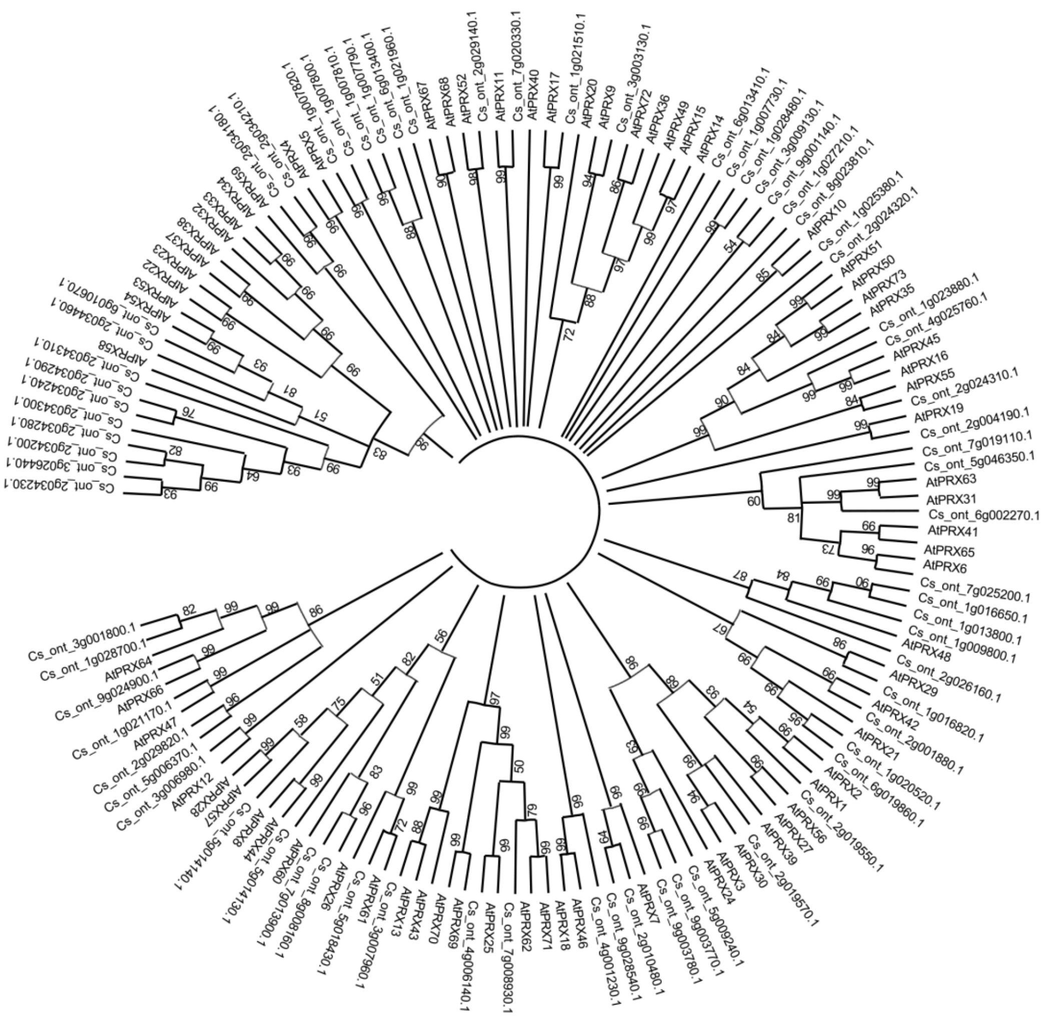

Figure S8. Phylogenetic analysis of the protein sequences of type III peroxidases (PRX) from *A. thaliana* and *C. sinensis*. The tree was generated as in Supplementary Figure S7 (WAG model, 1,000 bootstraps).

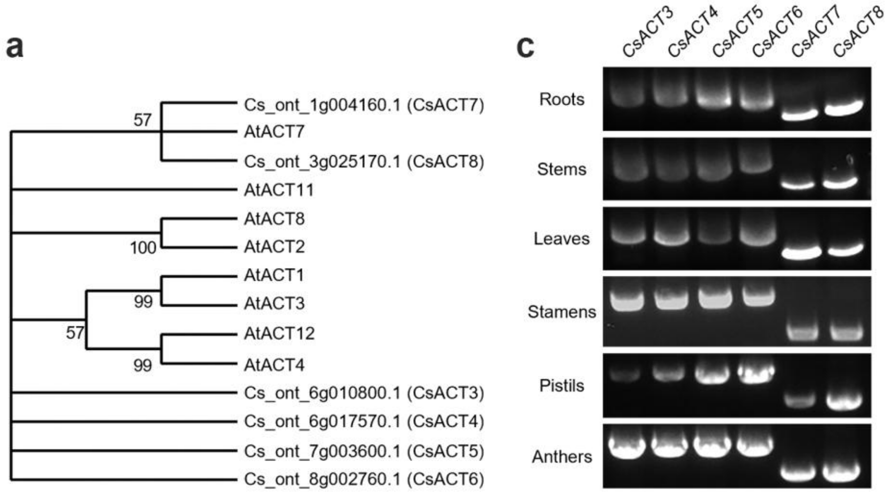

b

Name	Gene	Protein length (aa)	Superfamily
CsACT7	Cs_ont_1g004160.1	377	NBD_sugar-kinase_HSP70_actin superfamily
CsACT8	Cs_ont_3g025170.1	377	NBD_sugar-kinase_HSP70_actin superfamily
CsACT3	Cs_ont_6g010800.1	377	NBD_sugar-kinase_HSP70_actin superfamily
CsACT4	Cs_ont_6g017570.1	377	NBD_sugar-kinase_HSP71_actin superfamily
CsACT5	Cs_ont_7g003600.1	377	NBD_sugar-kinase_HSP73_actin superfamily
CsACT6	Cs_ont_8g002760.1	377	NBD_sugar-kinase_HSP70_actin superfamily

Figure S9. Genomic characterization and expression profiling of the *CsACT* gene family in *C. sinensis*. (a) Phylogenetic reconstruction of *actin* gene families. Tree was conducted using MEGA11 software on the amino acid sequences of 8 actin genes in *A. thaliana* (*AtACTs*) and 6 actin genes in *C. sinensis* (*CsACTs*). The analysis employed the MLmethod, utilizing the Le_Gascuel_2008 model for calculations. **(b)** Genomic organization of *CsACT* loci. Gene locus and translated amino acid lengths for each *CsACT* gene are annotated. **(c)** Tissue-specific expression patterns of *CsACT* genes. Semi-quantitative RT-PCR was performed using gene-specific primers (Table S1). Reproductive tissues analyzed include stamens, pistils, and pollen-containing anthers. Vegetative tissues: roots, stems, and leaves.

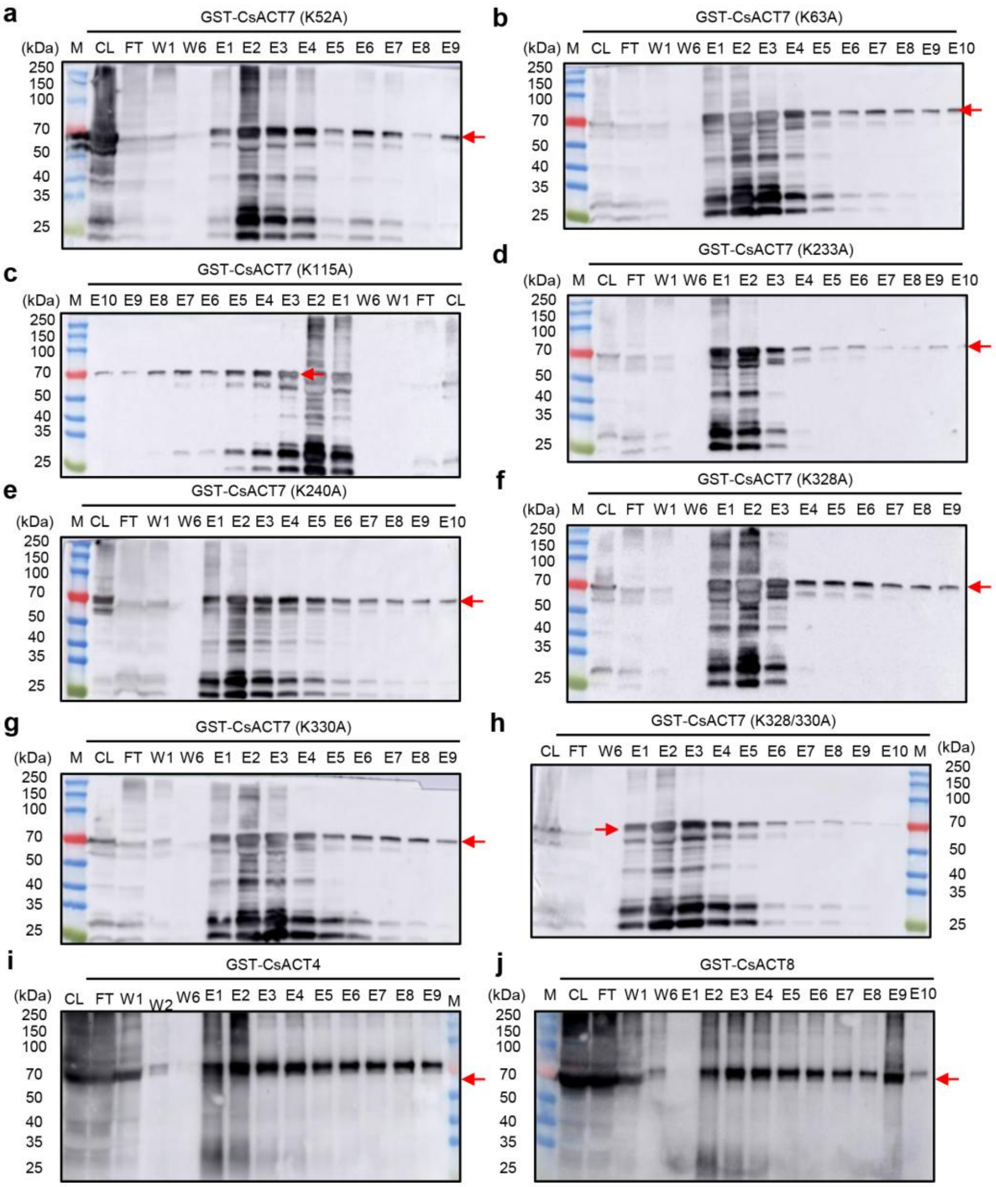

Figure S10. Purification results of GST-CsACT variants. Comprehensive quantitative ubiquitination proteomics analysis indicated increased ubiquitination levels of CsACT7 at K52, K63, K115, K233, K240, K328, and K330. Site-directed mutagenesis was employed to substitute the lysine (K) with alanine (A). **(a-j)** Protein purification results of the recombinant GST-CsACT7 single mutants (**a**, K52A; **b**, K63A; **c**, K115A; **d**, K233A; **e**, K240A; **f**, K328A; **g**, K330A), the GST-CsACT7 double mutant (**h**, K328/330A), GST-CsACT4 **(i)** and GST-CsACT8 **(j)** were assessed using WB with anti-GST antibody. M: marker; CL: cell lysate; FT: flow-through; W: wash; E: elution; Red arrow: GST-CsACT7 variants.

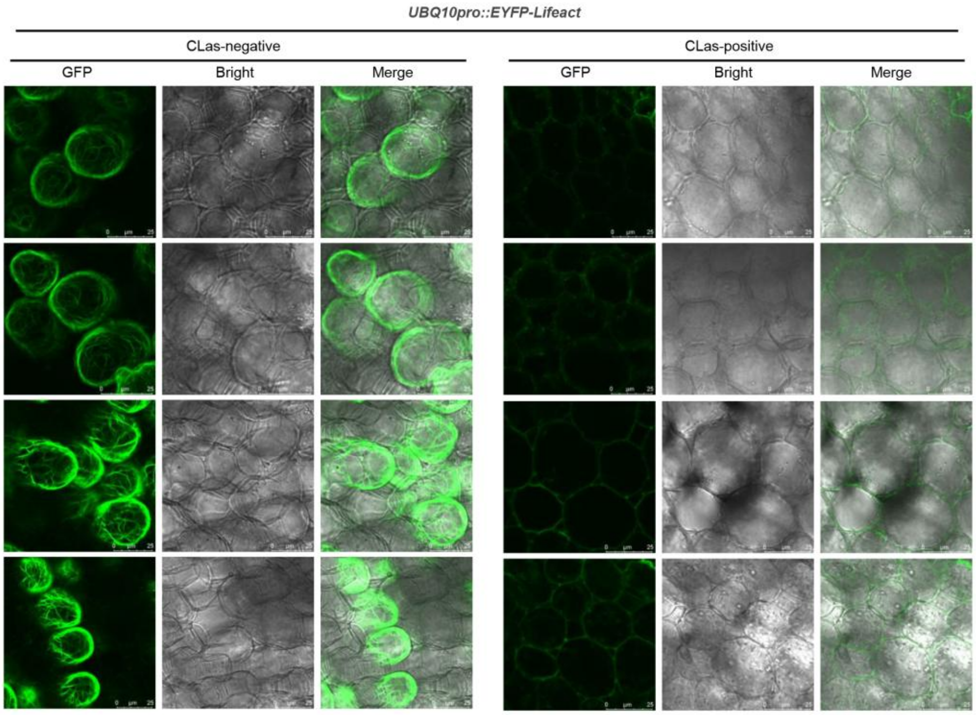

Figure S11. Degradation of the actin cytoskeleton under CLas infection. More images described in Figure 6(m) were demonstrated here.

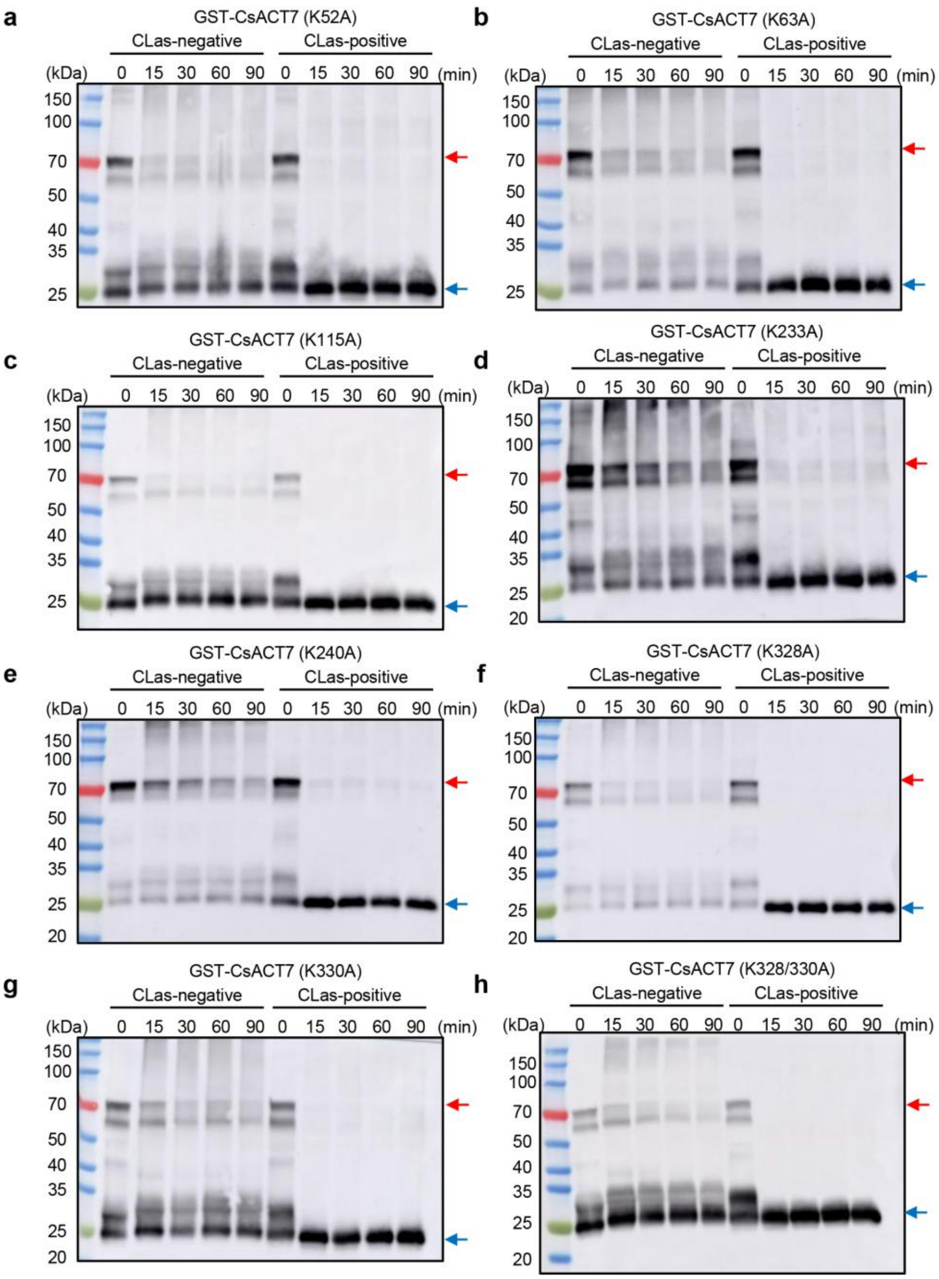

Figure S12. Degradation of GST-CsACT7 under CLas stress is independent of individual modification sites or the K328/K330 double sites. (a-h) The recombinant GST-CsACT7 single mutants (**a**, K52A; **b**, K63A; **c**, K115A; **d**, K233A; **e**, K240A; **f**, K328A; **g**, K330A) and the double mutant (**h**, K328/330A) were incubated with protein crude extracts from both HLB-infected and uninfected Newhall leaves at 25°C for designated time. Variations in GST-CsACT7 protein levels were assessed using WB with anti-GST antibody, as the actin antibody effectively recognized actins in the sweet orange leaf extracts. Due to the uneven distribution of CLas within a citrus tree, the bacterial titer of CLas in each leaf was individually confirmed using qPCR. Experiments were repeated at least twice with consistent results. Blue arrow: GST; Red arrow: GST-CsACT7 variants.

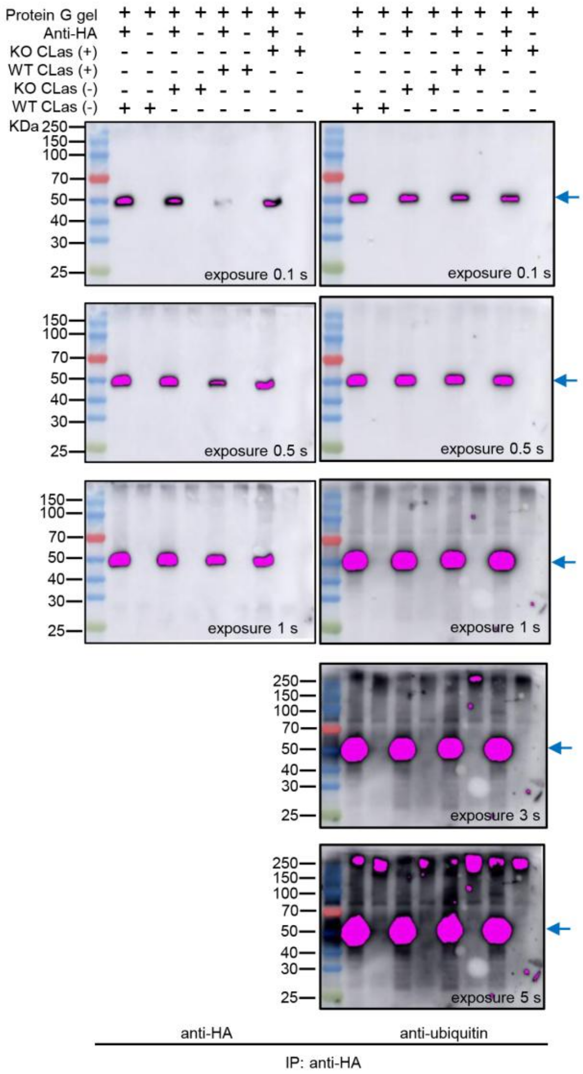

Figure S13. Full exposure time series for the immunoblot analysis shown in Figure 7D. Immunoprecipitated proteins were detected by immunoblotting with anti-HA and anti-ubiquitin antibodies, respectively. To comprehensively display the dynamic range of the signals, images from multiple exposure times are provided. For the anti-HA blot, images were acquired at exposure times of 0.1 s, 0.5 s, and 1 s. For the anti-ubiquitin blot, an extended exposure series of 0.1 s, 0.5 s, 1 s, 3 s, and 5 s was captured to visualize the high-molecular-weight polyubiquitination smears. As acknowledged in the main text, the 0.1 s exposure image of the anti-HA blot and the 5 s exposure image of the anti-ubiquitin blot are reused as representative panels in Figure 7d. The blue arrow indicates the unmodified CsACT7-3HA or CsACT7-KO-3HA monomers.

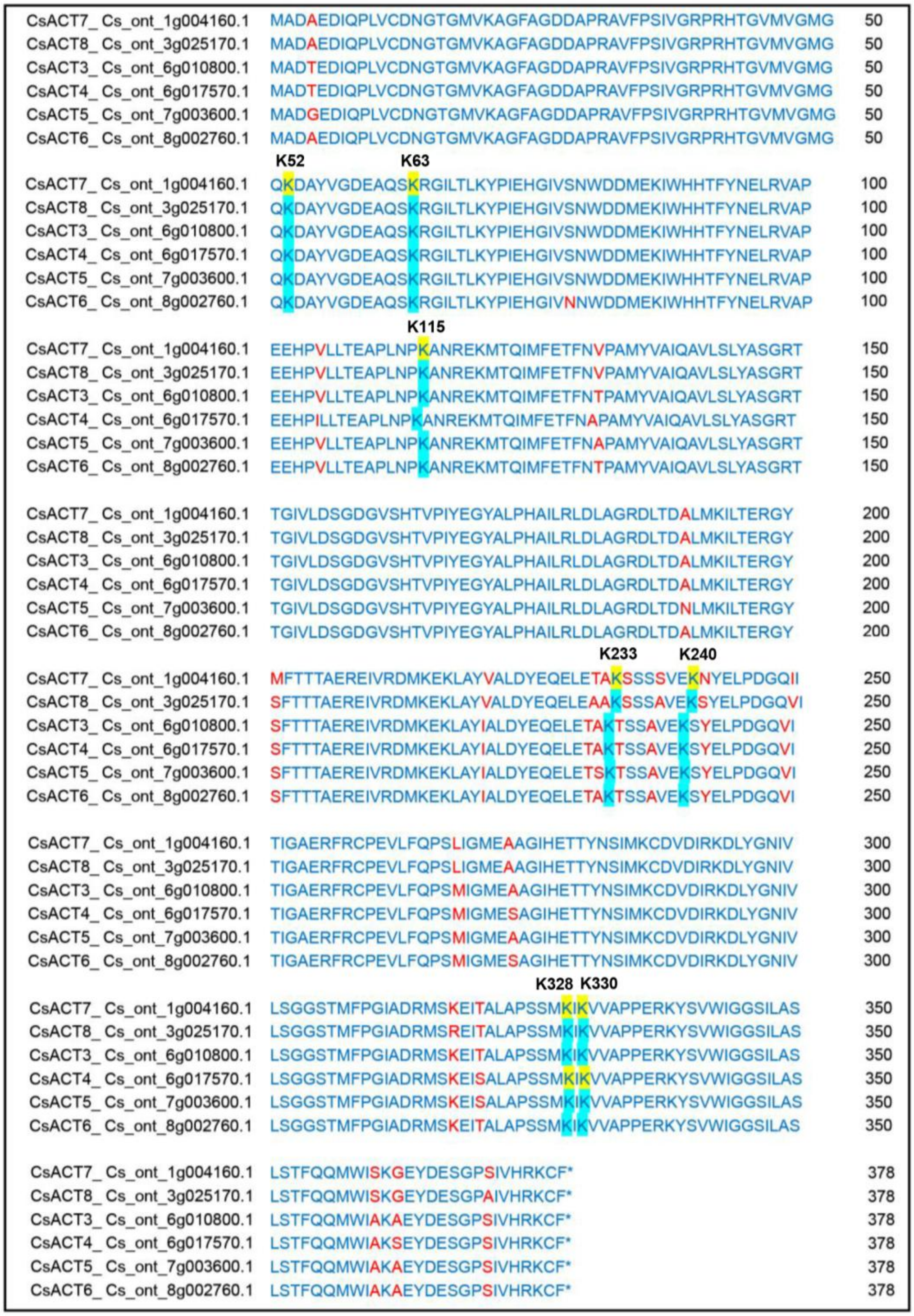

Figure S14. Multiple sequence alignment analysis of Citrus sinensis actin (CsACT) family proteins. Amino acid sequences of sweet orange (*C. sinensis*) actin isoforms were aligned using the ClustalW algorithm in MEGA11 software. Mutated amino acids are highlighted in red. Ubiquitination-modified sites identified as upregulated in CsACT4 and CsACT7 through quantitative ubiquitinomic profiling are marked in yellow. Corresponding lysine residues conserved in other sweet orange actin isoforms are annotated in blue.

Table S1. Primers used in this study.

Name	Sequence
CsActin7_RT_F-1	CCCTAGTATTGTTGGTAGGC
CsActin7_RT_R-1	TCCCAGTTGCTTACAATACC
GW_CsActin7_F	GGGGACAAGTTTGTACAAAAAAGCAGGCTTCATGGCCGATGCTGAGGATATTCAA
GW_CsActin7_R	GGGCACCACTTTGTACAAGAAAGCTGGGTCGAAGCATTTCCTGTGGACAATGGATG
pDONR207_F	TCGCGTTAACGCTAGCATGGATCTC
pDONR207_R	TGTAACATCAGAGATTTTGAGACACGG
attB1_F	ACAAGTTTGTACAAAAAAGCAGGCT
attB2_R	ACCACTTTGTACAAGAAAGCTGGGT
CsActin7_K52A_F	GGTTGGAATGGGGCSGGCTGATGCCTATGTTGGTG
CsActin7_K52A_R	CSCCSACSTAGGCSTCSGCCTGCCCCSTTCCSACC
CsActin7_K63A_F	TGATGAGGCCCSATCGGCTAGAGGTATTCTTACTT
CsActin7_K63A_R	AAGTAAGAATACCTCTAGCCGATTGGGCCTCSTCS
CsActin7_K115A_F	GGCSCCTCTTAACCCCGCTGCCSACSGAGAGAAGA
CsActin7_K115A_R	TCTTCTCTCTGTTGGCSGCGGGGTTAAGAGGTGCC
CsActin7_K233A_F	AGAACTTGAGACTGCCGCTAGCSGCTCSTCTGTTG
CsActin7_K233A_R	CSACSGATGAGCTGCTAGCGGCSGTCTCSAGTTCT
CsActin7_K240A_F	CSGCTCSTCTGTTGAGGCTAACTATGAGCTGCCTG
CsActin7_K240A_R	CSGGCSGCTCSTAGTTAGCCTCSACSGATGAGCTG
CsActin7_K328A_F-2	GCTCCSAGCSGCSTGGCTATCSAGGTCGTGGCT
CsActin7_K328A_R-2	AGCCSCGACCTTGATAGCCSTGCTGCTTGGAGC
CsActin7_K330A_F-2	AGCSGCSTGAAGATCGCTGTCGTGGCTCCSCCS
CsActin7_K330A_R-2	TGGTGGAGCCSCGACSGCGATCTTCSTGCTGCT
CsCstalase_RT_F-1	CTTCCGTCSACTACTCSCSA
CsCstalase_RT_R-1	ACSGTCSAGGAGTGATTGTC
CsActin4_RT_F-1	GCGTGGTATTTTGACTTTGA
CsActin4_RT_R-1	GCTTCSGTGAGGAGAATAGG
CsGAPDH_RT_F	GCTCTTCSGGGAAATGGTAA
CsGAPDH_RT_R	AGCSATGACTGAATGGAAGT
RNRf	CSTGCTCCSTGAAGCTACCC
RNRr	GGAGCSTTTAACCCCSCGAA
CsACT_RT_F	CCGAGCGTGGTTATTCTTTC
CsACT_RT_R	CTGAGCTGGTCTTTGCTGTCT
CsACT3_F	ATGGCSGATACTGAGGATATTCSGCC
CsACT3_R	ATCSTATTCTGCCTTTGCGATCCSC
CsACT4_F	ATGGCSGATACCGAGGATATTCSGC
CsACT4_R	ATCSTACTCSGATTTGGCSATCCSCSTTTG
CsACT5_F	ATGGCSGACGGTGAGGATATTCSG
CsACT5_R	GTCSTACTCSGCCTTTGCSATCCSC
CsACT6_F	ATGGCSGACGCTGAGGATATTCS
CsACT6_R	ATCSTACTCSGCCTTCGCSATCCSC
CsACT7_F	ATGGCCGATGCTGAGGATATTCSAC
CsACT7_R	ATAGTTCTTCTCSACSGATGAGCTGCT
CsACT8_F	ATGGCTGATGCTGAGGATATTCSACC
CsACT8_R	ATAACTCTTCTCSACTGCSGAGCTGC

pGEX-4T-CsACT4-F	TGGATCCCCGGAATTCSTGGCSGATACCGAGGATATTCSGC
pGEX-4T-CsACT4-R	GATGCGGCCGCTCGAGTCSGAAGCSTTTCCTGTGCSCTATTGAAG
pGEX-4T-CsACT8-F	TGGATCCCCGGAATTCSTGGCTGATGCTGAGGATATTCSAC
pGEX-4T-CsACT8-R	GATGCGGCCGCTCGAGCTAAAAGCSTTTTCTGTGTACSATAGCTGGAC
35S_CsACT7_F	CTAGAGGATCCTCGAGATGGCCGATGCTGAGGATATTCS
35S_CsACT7_R	cgtatgggtaGTCGACGAAGCSTTTCCTGTGGACSATGGA

Dataset S1. Comprehensive proteomic profiling of CLas-infected Newhall sweet orange leaves.

Dataset S2. Comprehensive ubiquitinomic profiling of CLas-infected Newhall sweet orange leaves.

Dataset S3. Differentially expressed proteins (DEPs) in CLas-infected Newhall sweet orange leaves.

Dataset S4. Differentially ubiquitinated sites in CLas-infected Newhall sweet orange leaves.

Dataset S5. Functional annotation of differentially ubiquitinated proteins (DUPs) in CLas-infected Newhall sweet orange leaves.

Dataset S6. List of the lysine ubiquitination sites in the DUPs in CLas-infected Newhall sweet orange leaves.

Dataset S7. GO enrichment analysis results for up- and down- regulated proteins in the Proteome.

Dataset S8. GO enrichment analysis results for up- and down- ubiquitinated proteins in the Ubiquitinome.

Dataset S9. Common protein list for venn diagrams between DEPs and DUPs in CLas-infected Newhall sweet orange leaves.

Dataset S10. GO and KEGG pathway enrichment analysis results for proteins with up expression and up ubiquitination.

Dataset S11. GO and KEGG pathway enrichment analysis results for proteins with down expression and up ubiquitination.

Dataset S12. Proteins identified across transcriptomic, proteomic, and ubiquitinomic analyses in CLas-infected Newhall sweet orange leaves.

Dataset S13. Motif-x analysis results.

Dateset S14. GO-Based functional enrichment characterization of DUPs with conserved Kub motifs.

Dataset S15. Motif classification of ubiquitinated sites in H_2O_2 scavengers and CsACTs in Newhall leaves after CLas-infection.

Dataset S16. Genes involved in H_2O_2 scavenging in *A. thaliana* and *C. sinensis*.

Dataset S17. Functional prediction of CLas proteins containing Sec secretion signals and other predicted virulence factors.
